# Interrupted DNA and Slow Silver Cluster Luminescence

**DOI:** 10.1021/acs.jpcc.3c01050

**Published:** 2023-05-31

**Authors:** David Lewis, Caleb Setzler, Peter M. Goodwin, Kirsten Thomas, Makayla Branham, Caleb A. Arrington, Jeffrey T. Petty

**Affiliations:** † Department of Chemistry, Furman University, Greenville, South Carolina 29163, United States; ‡ Center for Integrated Nanotechnologies, Mail Stop K771, Los Alamos National Laboratory, Los Alamos, New Mexico 87545, United States; § Department of Chemistry, Wofford College, Spartanburg, South Carolina 29303, United States

## Abstract

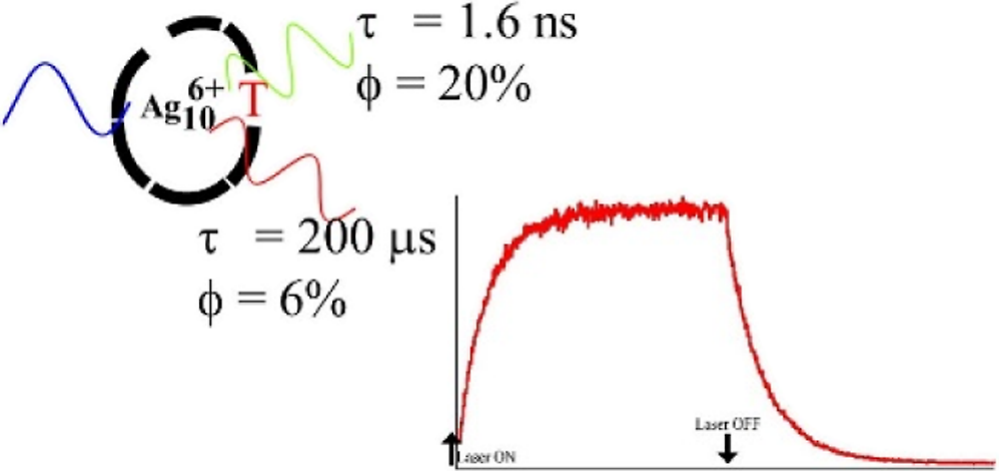

A DNA–silver cluster conjugate is a hierarchical chromophore with a partly reduced silver core embedded within the DNA nucleobases that are covalently linked by the phosphodiester backbone. Specific sites within a polymeric DNA can be targeted to spectrally tune the silver cluster. Here, the repeated (C_2_A)_6_ strand is interrupted with a thymine, and the resulting (C_2_A)_2_-T-(C_2_A)_4_ forms only Ag_10_^6+^, a chromophore with both prompt (∼1 ns) green and sustained (∼10^2^ μs) red luminescence. Thymine is an inert placeholder that can be removed, and the two fragments (C_2_A)_2_ and (C_2_A)_4_ also produce the same Ag_10_^6+^ adduct. In relation to (C_2_A)_2_T(C_2_A)_4_, the (C_2_A)_2_ + (C_2_A)_4_ pair is distinguished because the red Ag_10_^6+^ luminescence is ∼6× lower, relaxes ∼30% faster, and is quenched ∼2× faster with O_2_. These differences suggest that a specific break in the phosphodiester backbone can regulate how a contiguous vs broken scaffold wraps and better protects its cluster adduct.

## Introduction

Metals are integral to the structure and function of a DNA strand.^1,2^ For example, alkali-metal cations concentrate along the ionized phosphodiester backbone to regulate how proteins and drugs interact with a nucleic acid sequence.^3−5^ Also, transition metals coordinate with nucleobases and tightly regulate the secondary and tertiary structures of DNA and RNA.^6−8^ Orthogonal to these biological roles, metals can assemble with DNA to create new nanomaterials. Noble metal cations accumulate along the DNA and are reduced to form filamentous wires and other nanoscale electronic components.^9,10^ Transition metals such as Hg^2+^ crosslink nucleobases into extended arrays of metal-linked base pairs, which are used for electronic and materials applications.^11−13^ Here, we consider Ag^+^ that are collected within a DNA and are then chemically reduced to form molecular silver chromophores.

The hallmark of silver molecules is strong, tunable fluorescence with sensitivities reaching the single-molecule limit.^14^ These metallic molecular chromophores efficiently emit because their valence electronic states are sparsely organized due to atomic 5s states that are weakly coupled with underlying core states.^15−22^ However, bare silver clusters are reactive and thus require a protective scaffold.^23−26^ DNA is one such host. When sequestered within an oligonucleotide, clusters do not agglomerate or tarnish and survive in challenging biological environments with high salt and protein concentrations.^15,22,27−31^ Beyond protecting its silver cargo, DNA can be synthetically modified to tune the spectra of its silver cluster adducts, and three approaches have been demonstrated. One, the DNA sequence can be chosen to create a specific cluster chromophore. An oligonucleotide creates a distinct coordination site because its nucleobases bind silver with different affinities.^32−35^ Two, the structure of a DNA host can be reversibly switched between single- and double-stranded states to toggle the cluster brightness. Base pairing disrupts nucleobase–silver coordination, and the cluster can reorganize to yield ∼10^3^-fold stronger emission.^36−38^ Three, a DNA can activate cluster emission when the pH is changed. Acidic heteroatoms in the nucleobases can be deprotonated, and these open coordination sites control the cluster structure.^38−41^ Here, we discuss how a single nucleotide reshapes the DNA coordination site and reveals a new luminescent feature from its silver cluster adduct.

The repeated sequence (C_2_A)_6_ selectively forms a Ag_10_^6+^ adduct, a Ag_4_^0^-based cluster with green emission.^42,43^ In these studies, the versatility of this template is leveraged. Prior studies showed that the adenines can be replaced by thymines and imidazoles, and these modified strands preserve the cluster size and charge while quenching the cluster emission.^44^ Furthermore, (C_2_A) sub-sequences can be added, and these larger strands conserve the Ag_4_^0^ chromophore while expanding the number of Ag^+^ adducts. In these studies, the repeated C_2_A motifs are interrupted with a thymine. We chose thymine because it is a poor ligand for silvers, and we target this site with other chemically neutral modifications and by breaking the phosphodiester backbone. In all cases, the strands preserve the strong, prompt green fluorescence while also developing longer lived red luminescence. We leverage this luminescence to characterize the cluster coordination site in this nanoscale and multidentate DNA ligand.^45,46^

## Methods

DNA-silver clusters were synthesized using four components: an oligonucleotide as the template, Ag^+^ as the cluster precursor, BH4^–^ as the reducing agent, and O_2_ to eliminate labile clusters.^47^ Dehydrated oligonucleotides (Integrated DNA Technologies) were hydrated in deionized water, and stock concentrations were measured using the nearest neighbor approximation.^2^ Oligonucleotides were diluted to 30 μM in 5 mM sodium cacodylate buffer (pH = 7) before adding an 8:1 relative amount of AgNO_3_. This pH 7 buffer maintains the protonation in the DNA and stabilizes the negatively charged phosphate backbone via Na^+^ ions. BH4^–^ was added next in a 12:1 relative ratio with the DNA. Solutions were mixed thoroughly and placed in a high-pressure reaction chamber (Parr) at 400 psi O_2_ for ∼1.5 h.

Spectroscopic measurements were carried out on a Cary 50 UV–vis spectrophotometer (Varian) and a Fluoromax-3 spectrofluorometer (Jobin-Yvon HORIBA). Fluorescence quantum yields were quantified using fluorescein (φ_F_ = 95%) and tris(bipyridine)ruthenium(II) chloride (φ_F_ = 4.2%) for the respective green and red luminescence from the cluster.^48,49^ Mass spectrometry was performed using a Q-TOF G2-S mass spectrometer (Waters). Samples were diluted with deionized water to ∼2 μM and were infused via a syringe pump operated at a flow rate of 20 μL/min. The spectra were collected in the negative ion mode with a capillary voltage of −2.7 kV, a sampling cone voltage of −15 V, an extraction cone voltage of 10 V, a cone gas flow of 45 L/h, and a desolvation gas flow of 450 L/h. The source temperature was 80 °C, and the desolvation temperature was 150 °C. Mass calibration was performed using aggregates of sodium iodide in the 400 < *m*/*z* < 2000 range. The spectra were analyzed using MassLynx V4.1.

Time-correlated single photon counting was achieved using an ∼150 ps (FWHM) pulsed 420 nm laser at 10 MHz. The laser light was vertically polarized, and the emission was collected at a right angle through a second polarizer set to the magic angle (55°). The excitation beam was attenuated to achieve less than 5 emissive photons per 100 laser pulses (<500 kHz), and this emission was passed through a 525/20 nm bandpass filter. The instrument response function was measured using colloidal silica and was convolved with exponential decays and fit to the fluorescence decay to yield fluorescence lifetimes (FluoFit, PicoQuant). The lifetimes are intensity-weighted averages using three exponentials.

Time-resolved fluorescence anisotropy measurements were conducted with our pulsed laser setup. Samples were excited with vertically polarized light, and vertical (*I*_VV_) and horizontal (*I*_VH_) decays were measured. These decays were convolved with the instrument response function (IRF) to yield anisotropy (*r*(*t*)) and rotational correlation times (τ_c_). In turn, τ_c_ was used to determine hydrodynamic radii with a modified Stokes–Einstein–Debye relation.

Longer lived luminescence was characterized using an ∼1 kHz modulated cw argon ion laser tuned to 457.9 nm. An acousto–optic modulator (AOM) was used in conjunction with an adjustable iris to capture 1st order diffraction, yielding a modulated excitation beam. The AOM was controlled with a fixed frequency driver (MT200-A0.5-VIS, AA Opto-Electronic) which received square wave inputs (0–1 V) from a function generator (33250A, Agilent). The pulse rise time was ∼0.1 μs, and the excitation beam was focused using a 10× objective (RMS10×, Thor-labs) to give a minimum beam radius of 3.5 μm within a 3 × 3 mm fluorescence cell. Green and red luminescence were isolated with 525/20 and 694/44 nm bandpass filters, respectively, before arriving at an avalanche photodiode detector (APD) (SPD-050, Micro Photon Devices) positioned at 90° relative to the excitation path. The arriving photons were converted to transistor to transistor logic (TTL) pulses. For each duty cycle of the laser, multiple TTL pulses were histogrammed by a timer card (PCI-6612, National Instruments) with a clock tick rate of 100 MHz. Luminescence decays were plotted in LabVIEW and were fitted with single exponentials in IGOR.

Fluorescence correlation spectroscopy studies were conducted as discussed earlier.^39,50^ A 428 nm laser (RMPC Laser) was coupled into a single-mode optical fiber to produce a Gaussian beam profile (OZ Optics). The collimated beam at an average power of ∼40 μW was coupled into an inverted microscope using a 470 nm long pass dichroic (Semrock). The laser partially filled the back aperture of a 63 × 1.2 NA water immersion microscope objective (Zeiss). The emission was collected in an epi-illumination geometry, spectrally filtered by a 550/80 nm bandpass filter, and spatially filtered using a 75 μm diameter pinhole at the image plane of the microscope. The emission was evenly divided onto two actively quenched single-photon counting avalanche photodiode detectors (PDM, Micro Photon Devices). The TTL pulses from the detectors were cross-correlated to minimize artifacts due to detector after-pulsing and dead time (HydraHarp 400, PicoQuant). Sample droplets were interrogated ∼20 μm above the coverslip (BK7, 160 μm thick). A 5 nM solution of rhodamine 110 (R0016, Chemodex) determined the dimensions of the probe volume using a 3-D Gaussian model.

Autocorrelation analysis of the fluorescence fluctuations [*G*(τ)] was resolved into diffusive [*g*_D_(τ)] and excited-state [*g*_ES_(τ)] contributions
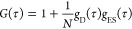
1where *g*_D_(τ) is based on a 3-D Gaussian model
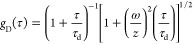
2and
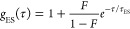
3

In these equations, τ is the lag time, *N* is the average number of fluorescent species in the probe volume, ω is the transverse radius of the probe volume, *z* is the height of the probe volume, τ_d_ is the lag time at which the autocorrelation amplitude has decayed to approximately one-half of its maximum value, *G*(0), *F* is the fractional occupancy of the dark state, and τ_ES_ is the net correlation time for dark state shelving. The aspect ratio *z*/ω was determined using rhodamine 110 and set to 6.7 for fitting. We used the occupancy (*N*) of the probe volume to determine the extinction coefficient of the clusters. These measurements determined the extinction coefficient of the (C_2_A)_6_–Ag10^6+^ chromophore. This DNA was used in lieu of (C_2_A)_2_-T-(C_2_A)_4_ because the shelving in the intermediate state diminished the fluorescence to preclude accurate quantitation. These studies were conducted over a 5-fold range of powers and concentrations. The resulting extinction coefficient was 137 (±19) × 10^3^ M^–1^ cm^–1^.

## Results

### Divided Strand

**Figure 1:**
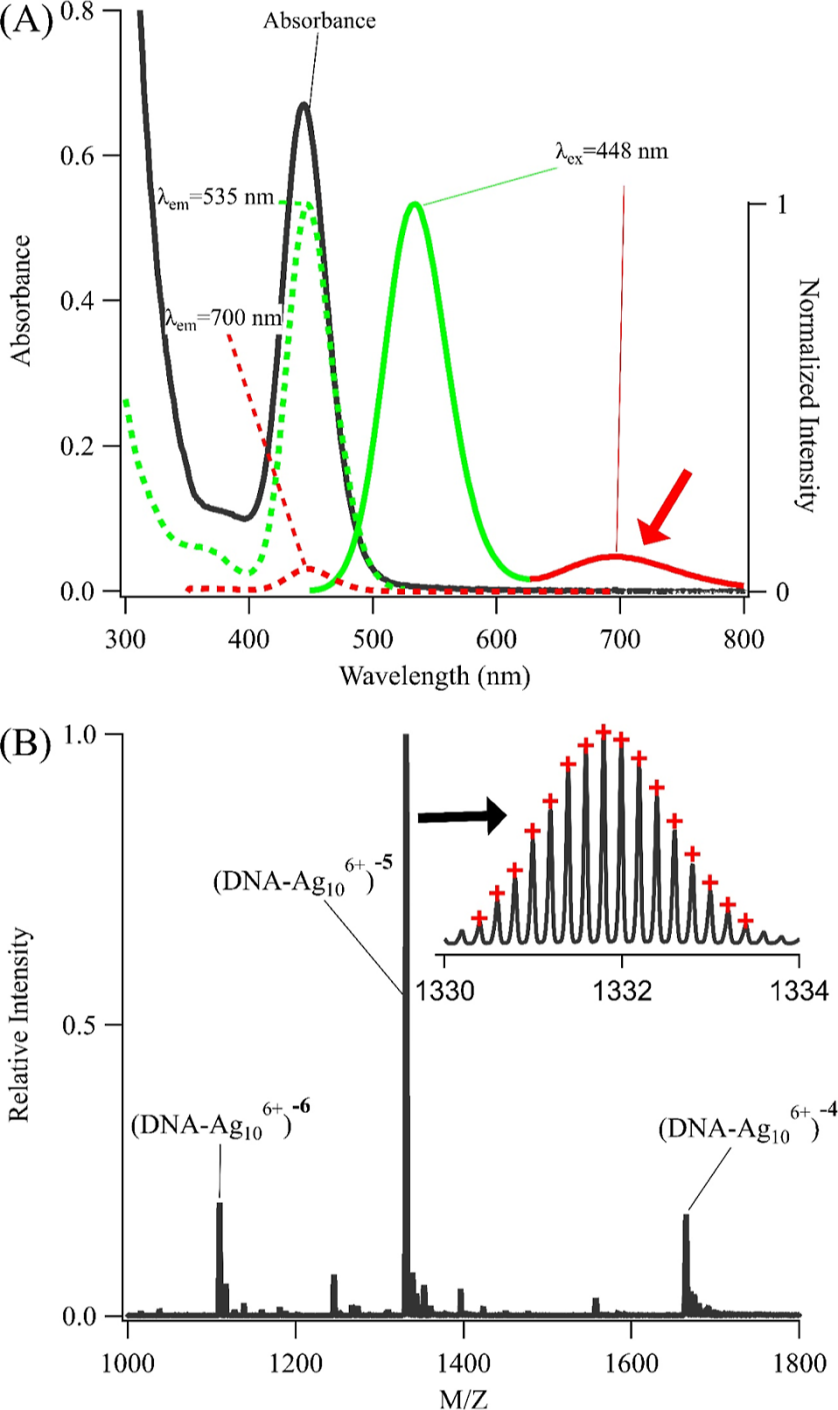
(A) Absorption (left axis, black) and excitation/emission (right axis) spectra of 2T4-Ag_10_^6+^. Excitation spectra with λ_ex_ = 448 nm (green and red dashed lines) yields both green emission and red luminescence (highlighted with red arrow). (B) Figure 2: Mass:charge spectrum of the −6, −5, and −4 charged ions of 2T4-Ag_10_^6+^. (Inset) Isotopologue distribution for the (DNA-Ag_10_^6+^)^5–^ complex is reproduced with a precision of 3.1 ± 0.6 ppm (red dots) for the three ions. Low abundance peaks are due to Ag^+^ and Ag_6_^4+^ adducts with 2T4 (Table S3).

(C_2_A)_6_ selectively forms the green-emitting Ag_10_^6+^, but variants reveal an additional luminescent band (Figure 1). This repeated sequence was systematically interrupted with a thymine, which was chosen because it is a poor ligand for silver.^35,39^ The five derivatives are denoted *x*T*y*, where *x* and *y* denote the number of C_2_A sub-sequences before and after the thymine, respectively.^35,39,51^ For example, 2T4 is CCA CCA-T-CCA CCA CCA CCA with 2 C_2_A before and 4 C_2_A after the thymine. These five strands preserve key elements of their parent (C_2_A)_6_ by developing chromophores with λ_abs_ = 435–444 nm and λ_em_ = 527–536 nm, and each is distinctive partly because of the 5′→3′ DNA polarity (Figures 1A and S1 and Table S1).^52^ Only a specific silver cluster chromophore is synthesized, as suggested by the consistent absorption maximum from 2–10 Ag/DNA and by a consistent emission maximum using λ_ex_ = 400–500 nm (Figure S2).^53−55^ Furthermore, these chromophores are like other DNA-bound silver molecules with ∼7–26% fluorescence quantum yields and 1.4–2.2 ns fluorescence lifetimes.^20,21,34^ These similarities suggest that thymine is a neutral substitution, but it indirectly alters the silver cluster adduct because pronounced red luminescence develops alongside the dominant green emission (Figure 1A, red arrow). This low-energy luminescence is not only spectrally but also kinetically distinct with an ∼3× lower quantum yield (6 vs 20%) and an ∼10^5^× longer lifetime (200 μs vs 1.6 ns) in relation to the green emission, as subsequently discussed.

The role of the added thymine was probed by synthetically targeting this site (Figure S1). In one approach, the thymine was replaced by uracil, which was then enzymatically excised.^56^ The resulting strand had an abasic site along the intact phosphodiester backbone. Equivalently, the entire thymine nucleotide was replaced with a triethylene glycol that covalently linked the (C_2_A)_2_ and (C_2_A)_4_ segments.^56^ Relative to 2T4, both derivatives yielded the same cluster with similar spectra. Furthermore, this seemingly dead site could be completely removed. The thymine nucleotide was eliminated to break 2T4 into (C_2_A)_2_ and (C_2_A)_4_ fragments (2 + 4, Table 1). This pair also yielded a cluster that emitted at both 530 and 690 nm with λ_ex_ = 445 nm, so a chemical or physical discontinuity in the (C_2_A)_6_ polymer was correlated with red luminescence (Figure S3). A 2 + 4 heterodimer was supported by the optical spectra (Figure S4). Mixed vs individual strands yielded distinct spectra, and the relative amounts of the two strands controlled the yield of the silver cluster adduct.^56^ Furthermore, the 2 + 4/and 2T4/cluster conjugates had similar global structures. When a relatively large DNA host tumbles, polarized emission from the embedded silver chromophore dephases.^57^ The resulting rotational correlation times gave hydrodynamic radii of 12.9 ± 0.2 and 13.2 ± 0.2 Å for the 2 + 4 and 2T4 complexes, respectively. These similar sizes suggest that (C_2_A)_2_ and (C_2_A)_4_ assembled and recreated the binding site in the contiguous 2T4. Both complexes were more compact than the 2T4 alone, whose larger hydrodynamic radius of 14.7 ± 0.4 Å suggests that the apo-DNA is unstructured.^58^ This size difference in the DNA strands suggests that silvers contract and assemble their DNA hosts via silver-nucleobase crosslinks.^44^ Thus, irrespective of how the thymine site is modified, the different DNA scaffolds all form the same dual-emission chromophore, whose formula was derived via mass spectrometry.

**Table 1:** Photophysical Measurements for 2T4/Ag_10_^6+^ and 2 + 4/Ag_10_^6+^^a^

sequence	φ_F_	τ_F_ (ns)	φ_C_	φ_I_	τ_I_ (μs)
**2T4**	0.20 ± 0.01	1.57 ± 0.03	0.07 ± 0.01	0.06 ± 0.03	200 ± 1
**2 + 4**	0.08 ± 0.01	1.3 ± 0.3	0.055 ± 0.004	0.01 ± 0.01	140 ± 1

a φ_F_ and τ_F_ are the quantum yield and lifetime, respectively, of the green fluorescence, φ_C_ is the S_1_ → I crossing efficiency, and φ_I_ and τ_I_ are the quantum yield and lifetime, respectively, of the red luminescence.

### Partially Reduced Cluster

DNA-bound silver clusters are chromophores because of their Ag^0^, but these are typically also partnered with Ag^+^.^15,17,42^ The net cluster sizes and oxidation states were measured via electrospray ionization mass spectrometry. As DNA-silver complexes transitioned from solution to gas phases, they gathered H^+^ from solution to form a range of partially neutralized ions.^59^ The 2T4-cluster complex had three ions with net −6, −5, and −4 charges (Figure 1B).^60−62^ After accounting for their charges, the theoretically fully protonated versions of these ions each had an average mass of 6664 amu, which most closely aligns with one 2T4 strand having 10 silvers, as with (C_2_A)_6_.^44^ However, the mass is ∼6 amu lower, and this deficit arises because the cluster was a mixture of Ag^0^ and Ag^+^. Ag^+^ adducts partly neutralize the net DNA charge and thereby reduce the number of phosphate-bound H^+^. These missing H^+^ were counted via the fine structure of the mass spectral peaks (Figure 1B, inset and Table S1). A given ion has a natural distribution of isotopes, and the pattern of isotopologue peaks is prescribed by the molecular formula.^61,63^ For example, the isotopologue masses and intensities of the −5 charged ion of the Ag_10_^6+^-labeled 2T4 are replicated by the formula [C_178_H_219_N_68_O_107_P_18_(Ag_10_^6+^)]^5–^, which has 11 fewer H^+^ than the fully protonated strand (Figure 1B). Five are absent because the overall complex has a −5 charge, so six are missing because the cluster has 6 Ag^+^. Alternate formulas with ±1H^+^ predict markedly different distributions with larger standard deviations and thus substantiate the loss of 11 H^+^ (Figure S5).^64^ The −6 and −4 charged ions also lacked 12 and 10 H^+^, respectively, relative to their unligated and fully protonated counterparts, so these also have 6 Ag^+^ (Table S2). Charges for gaseous DNA–silver complexes match the oxidation states of corresponding solution complexes, so these mass spectra indicate that Ag_10_^6+^ with 4 Ag^0^ is the chromophore.^42,65^

The (C_2_A)_2_ + (C_2_A)_4_ pair also forms the same silver molecule as 2T4, and this complex was identified by combining mass spectrometry and photochemistry studies. Ternary (C_2_A)_2_ + (C_2_A)_4_ + Ag_10_^6+^ complexes with net −4 and −5 charges were found, but other silver–DNA complexes also formed (Figure S6). So, the 2 + 4/Ag_10_^6+^ chromophore was distinguished by not only its mass but also its photochemistry. Silver clusters can be photolabile, and two changes resulted when the heterodimer/cluster chromophore was irradiated for an extended period at 428 nm–the λ_440_ absorbance peak was eliminated and the (C_2_A)_2_/(C_2_A)_4_/Ag_10_^6+^ complex in the mass spectra was destroyed (Figure S6, inset).^56,66^ The other peaks in the mass spectra remained, suggesting that photoexcitation specifically decomposed the Ag_10_^6+^ chromophore. In summary, the preceding optical and mass spectra show that both 2T4 and 2 + 4 formed a single Ag_10_^6+^ chromophore, and we now discuss its electronic structure and long-lived red luminescence.

### Slow Fluorescence and the Intermediate State

**Figure 2:**
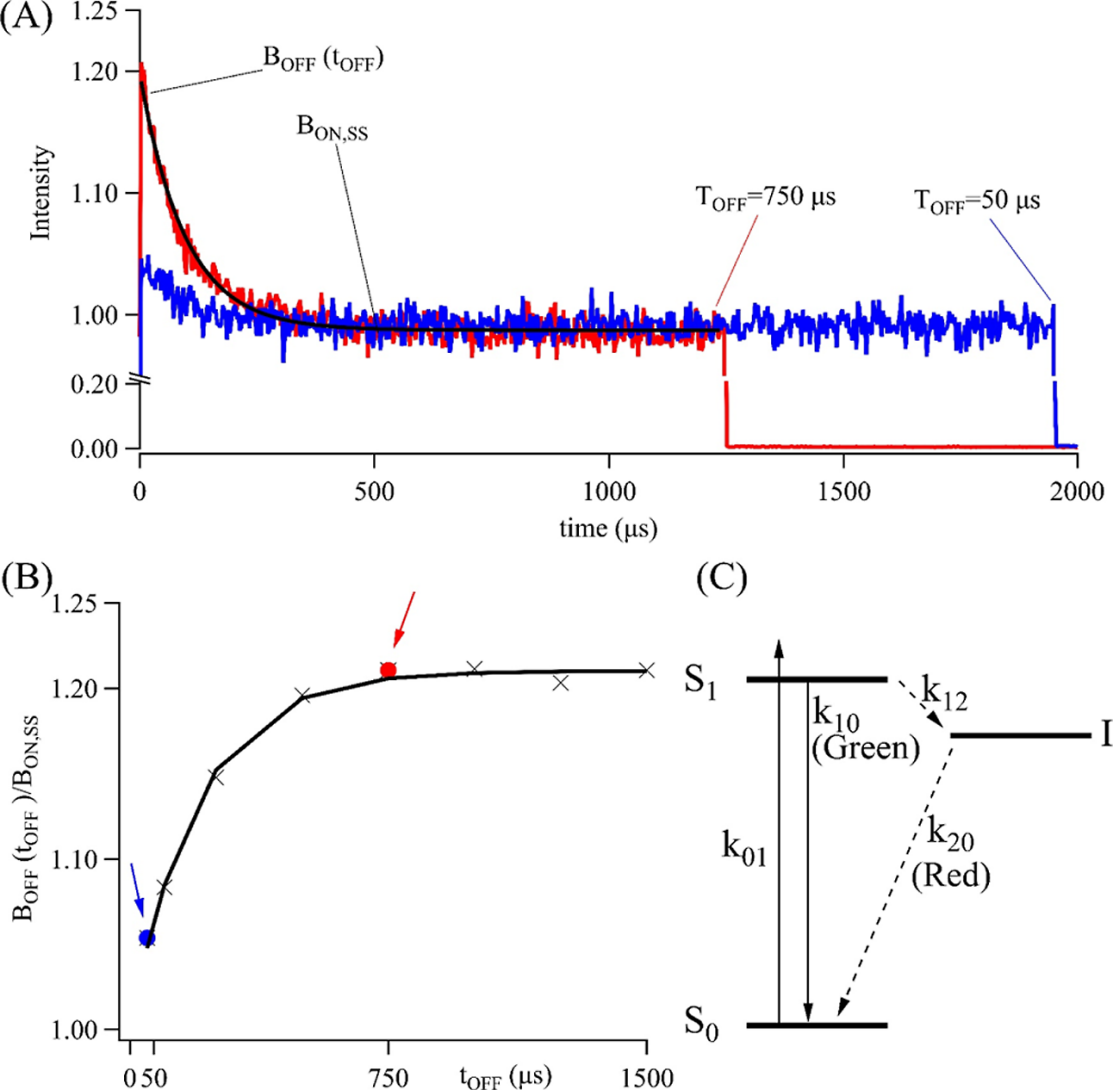
(A) Green fluorescence decay when 2T4-Ag_10_^6+^ is excited at 457 nm at 500 Hz. The fluorescence decays when the laser is on with *t*_OFF_ = 750 μs (red) and 50 μs (blue). Exponential fits (black) determined the amplitude [*B*_OFF_(*t*_OFF_)] and baseline (*B*_ON,SS_). The dynamic range of the decay is limited because the bright state population is large and is only slightly perturbed by I. (B) Normalized intensities were fit with eq 8 to determine *k*_ON_ = 1084 ± 47 Hz and *k*_OFF_ = 5150 ± 250 Hz. The red and blue arrows indicate the respective decays in (A). (C) Electronic state model for 2T4/Ag_10_^6+^ with ground (S_0_), emissive (S_1_), and intermediate (I) states and with connecting rate constants. Dashed lines emphasize relatively slow transitions to and from I.

Like other Ag_4_^0^-based clusters, the DNA-Ag_10_^6+^ chromophores produce strong, prompt green emission, so the long-lived red luminescence is distinctive. Both transitions share a common λ_ex_ = 440 nm, so this single excitation route out of the ground S_0_ state supports two excited states—a higher-lying S_1_ state that feeds its neighboring intermediate I state (Figure 2C). Linked S_1_ and I states are also supported by two types of fluorescence kinetic studies. In one approach, the 535 emission intensity was monitored while varying the excitation irradiance (Figure S7). The emission rate increased linearly at low irradiances but was tempered at higher fluences with nonlinearity beginning at *I* ∼ 45 W/cm^2^. Using our measured absorption cross-section σ = 2 × 10^–16^ cm^2^/molecule (vide infra), the corresponding excitation rate *k*_01_ = σ *I* ∼20 kHz was ≳10^4^ × slower than the ∼600 MHz (τ_F_ = 1.6 ns) green emission rate. These distinct rates suggest that population in S_1_ can relax via two routes—directly and efficiently to S_0_ with green emission vs indirectly and slowly via I to produce the lower energy red luminescence. The latter S_1_ → I coupling is relatively weak and only becomes prominent at higher irradiances that are used in our subsequent experiments.

The link between the intermediate and singlet states was also studied by modulating the laser intensity and tracking the sluggish fluorescence changes. The three-level model can be simplified because I weakly couples with S_0_ and S_1_^46,67^
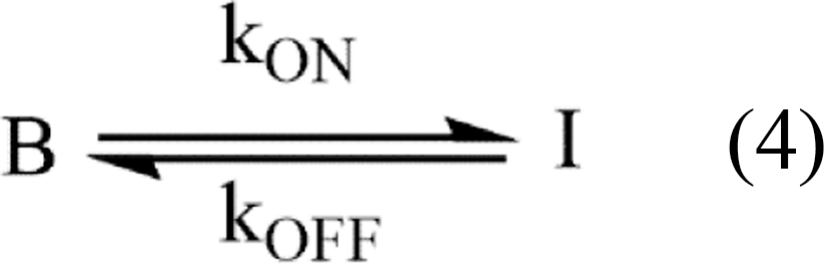
4

Here, B collectively represents the S_0_ and S_1_ populations that are strongly coupled due to short ∼ns lifetimes and rapid laser excitation. With strong laser irradiation, populations in these two states pre-equilibrate and reach a steady state faster than crossing to and from I. In relation to the three-level model, the rate constant *k*_ON_ describes excitation and crossing from S_0_ ⇌ S_1_ → I when the laser is on, while *k*_OFF_ describes I → S_0_ relaxation when the laser is off. This B ⇆ I equilibrium was shifted by modulating the laser intensity.

When the laser is on, the Bright state population (*B*_ON_) is initially high and then gradually decays because it shuttles to the I state^46^
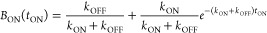
5where *t*_ON_ is the time the laser is on during its duty cycle. After an extended period (*t*_ON_ → ∞), the B and I populations equilibrate to a steady-state distribution *B*_ON,SS_
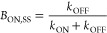
6

After the laser is turned off, the metastable I population reverts to S_0_^67^
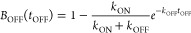
7where *B*_OFF_ is effectively the S_0_ population, as S_1_ clusters almost instantaneously relax. The time the laser is off during its duty cycle is *t*_OFF_, and longer off times allow more time for the metastable clusters to relax and thus increase the *B*_OFF_ population. After sufficient time (*t*_OFF_ = ∞), the population is fully restored to the ground state to give *B*_OFF_ = 1. The recovered *B*_OFF_ population was normalized using the steady-state *B*_ON_ population from eq 6
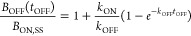
8

The parameters *B*_OFF_(*t*_OFF_) and *B*_ON,SS_ were derived from the slow fluorescence decay of DNA–Ag_10_^6+^ when the laser intensity was modulated (Figure 2A). The fluorescence intensities are strongest at the start of each cycle because the laser has been off and the S_0_ and thus the B state has been replenished during this dark period. Thus, *B*_OFF_(*t*_OFF_) is proportional to the initial amplitude of the exponential decay. When the duty cycle shrinks, the longer *t*_OFF_ allows greater recovery and higher initial intensities [compare red (*t*_OFF_ = 750 μs) and blue (*t*_OFF_ = 50 μs) traces and markers in Figure 2A,B]. After the laser has been on for a sufficient time, the fluorescence intensity plateaus and is proportional to *B*_ON,SS_. The ratio of *B*_OFF_(*t*_OFF_)/*B*_ON,SS_ was plotted vs *t*_OFF_ and fit using eq 8 to determine *k*_ON_ and *k*_OFF_. The resulting *k*_OFF_ depends on the host—this rate constant is ∼2× slower for 2T4/Ag_10_^6+^ vs 2 + 4/Ag_10_^6+^ (5.2 vs 10.3 kHz, Figure S8). Similar rate constants for other DNA-bound fluorophores suggest that long-lived, intermediate states may be common for these complexes.^68−70^ The derived *k*_ON_ was used to measure the S_1_ → I crossing efficiency in the three-level model (Figure 2C). This conversion is sequential with S_0_ → S_1_ excitation (*k*_01_) followed by S_1_ → I crossing (*k*_12_)
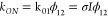
9where ϕ_12_ is the I → S_1_ crossing efficiency. A linear change in *k*_ON_ with the irradiance gives ϕ_12_ = 7 ± 1% for 2T4/Ag10^6+^ vs 5.5 ± 0.5% for 2 + 4/Ag10^6+^ (Figure S9). In summary, these fluorescence-based kinetic studies identify an intermediate I state that perturbs the emissive S_0_/S_1_ states.

### Slow Luminescence and the Intermediate State

**Figure 3:**
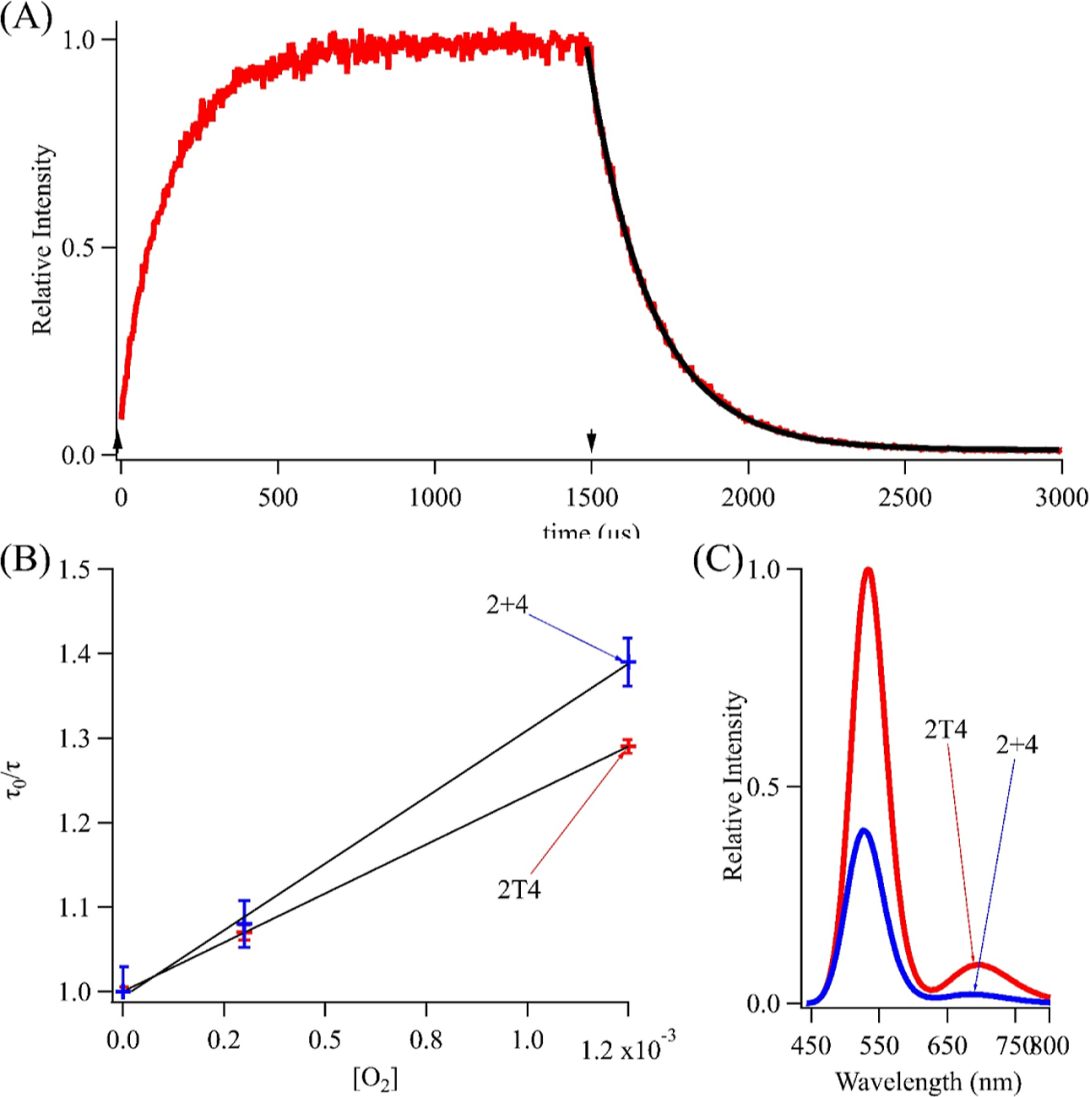
(A) Time evolution of red luminescence using a 333 Hz modulated laser with a 50% duty cycle and *t*_ON_ = 1500 μs. A single exponential fit (black) during the off period yielded *k*_OFF_ = 5.0 kHz. The small arrows indicate when the laser is on and off. (B) Stern–Volmer analysis derived from the near-infrared luminescence lifetimes without (τ_0_) and with (τ) oxygen ([O_2_]). The slope τ_0_*k*_SV_ from the linear fit yielded *k*_SV_ = 1.08 (±0.04) × 10^6^ M^–1^ s^–1^ for 2T4/Ag_10_^6+^ (red) and 2.1 (±0.1) × 10^6^ M^–1^ s^–1^ for 2 + 4/Ag_10_^6+^ (blue). (C) Luminescence spectra of 2T4/Ag_10_^6+^ (red) and 2 + 4/Ag_10_^6+^ (blue). The two solutions have matching absorbances (0.05) at their λ_max_.

A full picture of the B ⇆ I interconversion was developed using the red luminescence because it directly reports entry to and exit from the I state. When the laser is turned on, the red luminescence slowly builds to a plateau, and this growth is the reciprocal of the green emission decay (compare Figures 2A and 3A). Opposing growth and decay suggests that the I state is slowly fed via the higher lying S_1_ state, as supported by a common λ_ex_ for the red and green luminescence. When the laser is turned off, the red luminescence exponentially decays, as predicted by the first-order kinetic model (eq 4)
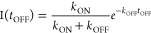
10

As the fluorescence decays to a steady state with the laser on, the red luminescence likewise decays with the laser off, both with the same *k*_OFF_ = 5.0 kHz (compare Figures 2A and 3A). Matching *k*_OFF_ from both the fluorescence and luminescence measurements suggests that only the S_0_ and I states are coupled with no additional relaxation paths. The *k*_off_ rate constants are constant over a 100× variation in the laser irradiance, which suggests that laser absorption by the transiently populated Intermediate state is limited (Figure S11).^71,72^

In relation to the rapid S_1_ → S_0_ transition (∼ns), the much slower I → S_0_ decay (10^2^ μs) opens a longer time window to study the cluster reactivity. Here, we consider quenching of the metastable DNA–Ag_10_^6+^ by O_2_.^46,57^ Oxygen concentrations were varied by saturating solutions with nitrogen, air, and oxygen at ambient pressure, and Stern–Volmer analysis yielded a dynamic quenching constant of 1.08 (±0.04) × 10^6^ M^–1^ s^–1^ for 2T4/Ag_10_^6+^ (Figure 3B). This is ∼10^4^× lower than the theoretical value 1.6 × 10^10^ M^–1^ s^–1^, derived from diffusion coefficients of 2 × 10^–5^ and 1.4 × 10^–6^ cm^2^/s for O_2_ and the DNA–Ag_10_^6+^ complex, respectively, and a collisional radius of 10 Å.^43,73,74^ The relatively lower observed rate constant supports a cluster that is embedded within a DNA matrix, but the cluster reactivity depends on the host.^74^ With the 2 + 4 pair, Ag_10_^6+^ reacts with O_2_ with a rate constant of 2.1 (±0.1) × 10^6^ M^–1^ s^–1^, ∼2-fold faster than for 2T4/Ag_10_^6+^. This faster rate suggests that the structural integrity of the phosphodiester backbone regulates how its cluster reacts with O_2_. This effect may be cluster specific, as other DNA-bound clusters are not sensitive to O_2_.^55^

### Kinetic Model

We now integrate our observations to model the slow fluorescence and luminescence kinetics.^46,67,75^ The S_0_ (ground), S_1_ (green emissive), and I (red luminescent) states are linked via elementary rate constants with respective subscripts 0, 1, and 2 (Figure 2C). The S_0_ → S_1_ excitation rate constant is *k*_01_ = σ*I*, and the absorption cross section σ was measured for (C_2_A)_6_–Ag_10_^6+^ in lieu of 2T4/Ag_10_^6+^. The parent (C_2_A)_6_–Ag_10_^6+^ fluorophore has similar absorption and fluorescence spectra as well as fluorescence lifetimes and quantum yields as 2T4/Ag_10_^6+^ (Table S1). Importantly, no long wavelength luminescence is observed for (C_2_A)_6_/Ag_10_^6+^, so it forms the intermediate state less efficiently than 2T4/Ag_10_^6+^.^44^ Therefore, the concentrations and hence extinction coefficient of this complex could be studied using fluorescence correlation spectroscopy because the high laser irradiances did not build up population in a long-lived intermediate state (Figure S10).^76^ These studies yielded an absorption cross section (σ) of 2 × 10^–16^ cm^2^/molecule (ε = 137,000 M^–1^ cm^–1^), in line with other DNA–silver cluster complexes, so this σ was used for both 2T4/Ag_10_^6+^ and 2 + 4/Ag_10_^6+^.^34,69,77^ Thus, using this σ for (C_2_A)_6_/Ag_10_^6+^ and our typical irradiance of 70 W/cm^2^ for 2T4/Ag10^6+^ gives *k*_01_ = 30 kHz.^50,56^ This rate constant exceeds the ∼20 kHz threshold where the DNA–silver chromophores are driven to the I state (Figure S7). The S_1_ → S_0_ relaxation rate constant *k*_10_ = 600 MHz is derived from the 1.6 ns fluorescence lifetime (Table 1). The I → S_0_ relaxation rate *k*_20_ is 5 kHz, which was derived from the decays of the green emission and near-infrared luminescence (Figures 2A and 3A). The crossing rate constant *k*_12_ = φ_12_*k*_10_ = 40 MHz is calculated from the φ_12_ = 7% quantum yield for S_1_ → I, consistent with prior results.^46,68,78^

**Figure 4:**
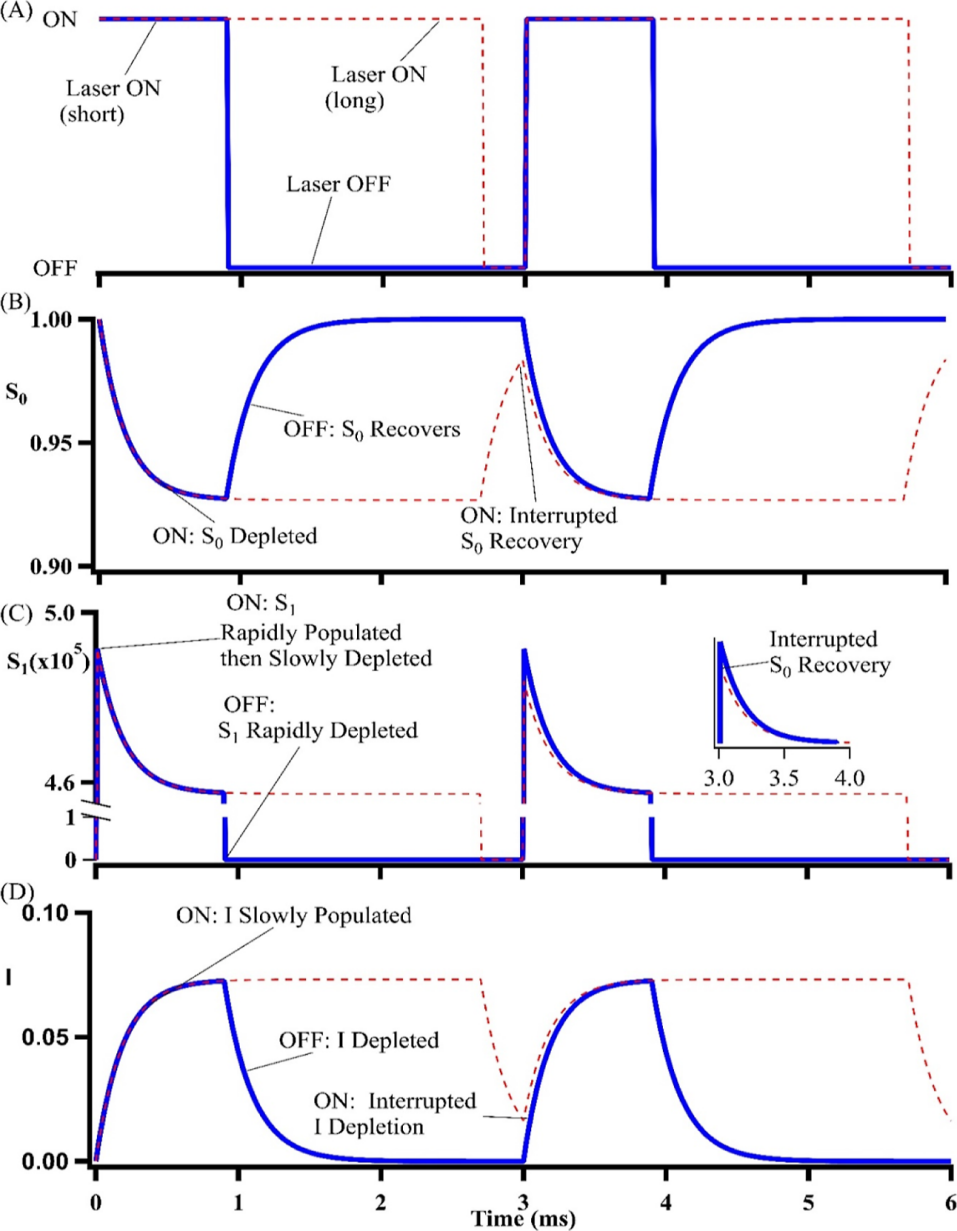
(A) Two laser excitation cycles at 33 Hz with *t*_OFF_ = 2100 (blue solid) and 30 (red dotted) μs. (B–D) Time evolution of the relative populations in S_0_, S_1_, and I, respectively. When the laser is on, the S_0_ population diminishes, the S_1_ population promptly jumps [*B*_OFF_(*t*_OFF_)] and then gradually decays, and the I population slowly grows. The steady-state distribution in S_1_ is *B*_ON,SS_. Note the break in the ordinate for (C) that emphasizes the limited dynamic range of the fluorescence decay. When the laser is turned off, the S_0_ population recovers, the S_1_ population abruptly drops, and the I population gradually decays. If the laser is turned back on too quickly, the recovery is interrupted (compare solid and dotted lines).

These experimental rate constants and the differential rate equations describe the time-dependent state populations in S_0_, S_1_, and I (Figure 4).^67,75^ When the laser is on, the DNA–silver clusters are rapidly driven from S_0_ to S_1_ but then gradually accumulate in the metastable I to yield a steady-state S_0_–S_1_–I distribution. When the laser is then turned off, the S_1_ clusters abruptly return to the ground state while their I counterparts more slowly drain down. This recovery depends on the duty cycle of the laser. If the laser is modulated with a short off time, the trapped population in I reduces the population in S_0_ and thus the initial fluorescence burst (compare solid and dotted lines in Figure 4 and compare with Figure 2A).

Further kinetic analysis highlights differences in the 2T4 and 2 + 4 scaffolds. 2T4/Ag10^6+^ has brighter and slower red luminescence relative to its 2 + 4 analogue; so the associated rate constants were decomposed into their radiative (*k*_r_) and nonradiative (*k*_nr_) contributions (Figure 3A,C).^79,80^ With 2T4/Ag_10_^6+^ vs 2 + 4/Ag_10_^6+^, *k*_r_ is ∼6× faster than *k*_nr_ [4.3 (±2.3) kHz vs 0.7 (±3.1) kHz]. Oppositely, with 2 + 4/Ag_10_^6+^ vs 2T4/Ag_10_^6+^, *k*_nr_ is ∼4.5× faster [5.9 (±1.4) kHz vs 1.3 (±1.3) kHz]. The differences are less distinct for the fluorescence with both 2T4 and 2 + 4 having similar nonradiative relaxation constants but have 3× different *k*_r_: [127 (±6) MHz and 48 (±7) MHz, respectively]. Thus, the long-lived red luminescence offers a distinct perspective of the DNA coordination sites.

## Discussion

DNA strands encode silver cluster spectra via their sequence, and the thymine in (C_2_A)_2_-T-(C_2_A)_4_ [2T4] is the basis of a dual-luminescent silver cluster. Like its parent (C_2_A)_6_, 2T4 develops a Ag_10_^6+^ chromophore with not only strong, short-lived green emission but also red luminescence with φ = 6% and τ = 200 μs. This long-lived luminescence is indirectly attributed to the thymine because its effect can be replicated with an abasic site and an ethylene glycol linker, also chemically inert DNA modifications. Thus, the thymine in 2T4 is a placeholder with flanking (C_2_A)_2_ and (C_2_A)_4_ tracts. Without the thymine, the (C_2_A)_2_ + (C_2_A)_4_ pair [2 + 4] assemble and also form the same dual-luminescent Ag10^6+^ with not only matching spectra but also similar hydrodynamic radii to 2T4/Ag_10_^6+^. The global structures of these 2 + 4/Ag_10_^6+^ and 2T4/Ag_10_^6+^ chromophores are smaller than the native and unstructured 2T4 oligonucleotide, so the (C_2_A)_2_ and (C_2_A)_4_ sub-sequences might innately associate via their shared Ag_10_^6+^ adduct. This assembly can be either intermolecular to form the 2 + 4 heterodimer or intramolecular to form the folded 2T4. In this folded structure, the thymine could be a chemically neutral hinge, so this (C_2_A)_2_ + (C_2_A)_4_ coordination site is peripherally guided by the DNA backbone.^81,82^

The targeted break in the phosphodiester backbone perturbs the Ag_10_^6+^ cluster and its red luminescence. The cluster luminescence is quenched ∼2-fold more efficiently by O_2_ in 2 + 4 vs 2T4. The breach in the phosphodiester backbone may expose the encapsulated cluster and thereby enhance quenching by O_2_, as exhibited by surface-bound and buried tryptophans in proteins.^83^ Besides its enhanced reactivity, the innate electronic relaxation of the Ag_10_^6+^ adduct is faster in the 2 + 4 scaffold due to a 6× drop in the measured luminescence quantum yield and a 30% decrease in the luminescence lifetime relative to 2T4/Ag10^6+^. These changes may be due to faster nonradiative decay out of the I state for 2 + 4. The contiguous and broken scaffolds are two extremes, and intermediate structures could be explored through bifunctional DNA strands. The cluster-coordinating (C_2_A)_2_ and (C_2_A)_4_ moieties could each be appended with sequences that hybridize with a common complementary strand.^36,37,56,84^ When the three strands collectively hybridize, (C_2_A)_2_ and (C_2_A)_4_ could be pulled together and thus positioned to form the Ag_10_^6+^ adduct. The supporting duplexes can be modified to control the relative positions, polarities, and coordination environments of their dangling 2 + 4 pair. These changes might then finely tune the cluster spectra.^85^

The sustained red luminescence has key characteristics of phosphorescence.^86^ It emanates from an intermediate I state that may be a triplet because it is ∼0.5 eV lower than its neighboring green emissive state.^86^ It is less efficient than the green fluorescence with an ∼3-fold lower quantum yield and ∼10^5^ slower decay time, so the associated S_1_ → I and I → S_0_ transitions may be spin-forbidden.^87^ Finally, it is partially quenched by oxygen, a neutral probe that penetrates the DNA host and transfers energy while conserving spin.^86^ Although phosphorescence can be efficiently quenched for organic chromophores, quenching can be inefficient for buried and protected chromophores.^57,88^ We propose that Ag_10_^6+^ is similarly shielded by its 2T4 and 2 + 4 hosts, as these wrap around the cluster. Protected environments favor metastable silver clusters. For example, Ag_4_^2+^ phosphoresces in the secluded cavities of zeolites with lifetimes of 1–100 μs.^89^ Likewise, DNA-bound silver clusters favor metastable states in polymer matrices and in deuterated solvents that limit vibrational relaxation.^14,90^ Theoretical calculations show that the efficiency of intersystem crossing increases ∼1000-fold from free to DNA-bound silver clusters, and these crossing rates could be tuned by the DNA sequence and structure.^34−36,87,91^

## Conclusions

A DNA host can be specifically modified to tune the spectrum of its silver cluster adduct. Here, the thymine in (C_2_A)_2_-T-(C_2_A)_4_ was targeted because it elicits long-lived red luminescence from its Ag_10_^6+^ adduct. Removing this thymine and clipping the phosphodiester backbone perturbs the coordination site, as evidenced by the reactivity and relaxation of the metastable Ag_10_^6+^. The impact of this broken DNA structure suggests that the phosphodiester backbone is an integral component in the framework for DNA-embedded silver cluster chromophores.

## Supporting Information Available

Absorption spectra of (C_2_A)_6_ and *x*T*y*-silver cluster chromophores; absorption spectra of 2T4 with differential equivalents of Ag^+^; absorption and excitation/emission spectra of 2 + 4/Ag_10_^6+^; absorption spectra of silver adducts with (C_2_A)_2_ alone, (C_2_A)_4_ alone, and (C_2_A)_4_ + different equivalents of (C_2_A)_2_; isotope model for the −5 charge state of 2T4/Ag_10_^6+^; mass:charge spectra of [2 + 4/Ag_10_^6+^]^−4^ before and after irradiation; fluorescence intensity vs irradiance for 2T4/Ag_10_^6+^; dependence of k_ON_ with the excitation rate *k*_01_; spectral and fluorescence parameters for *x*T*y* derivatives of (C_2_A)_6_; and mass:charge ESI data for 2T4/Ag_10_^6+^ and 2 + 4/Ag_10_^6+^ (PDF)
