# Supplementary material for: Interrupted DNA and Slow Silver Cluster Luminescence
Source: J Phys Chem C Nanomater Interfaces. 2023 May 31;127(22):10574–84. doi: 10.1021/acs.jpcc.3c01050 (PMC10258842; doi:10.1021/acs.jpcc.3c01050)
Supplement: Supplementary file 1 — jp3c01050_si_001.pdf [file jp3c01050_si_001.pdf]

An Interrupted DNA and Slow Silver Cluster Luminescence

David Lewis[†§] , Caleb Setzler[§], Peter Goodwin[ǁ], Kirsten Thomas[§], Makayla Branham[§], Caleb Arrington[‡], and Jeffrey Petty [†§*]

[§]Department of Chemistry, Furman University, Greenville, SC 29163, United States

[ǁ]Center for Integrated Nanotechnologies, Mail Stop K771, Los Alamos National Laboratory, Los Alamos, New Mexico 87545, United States

[‡]Department of Chemistry, Wofford College, Spartanburg, SC 29303, United States

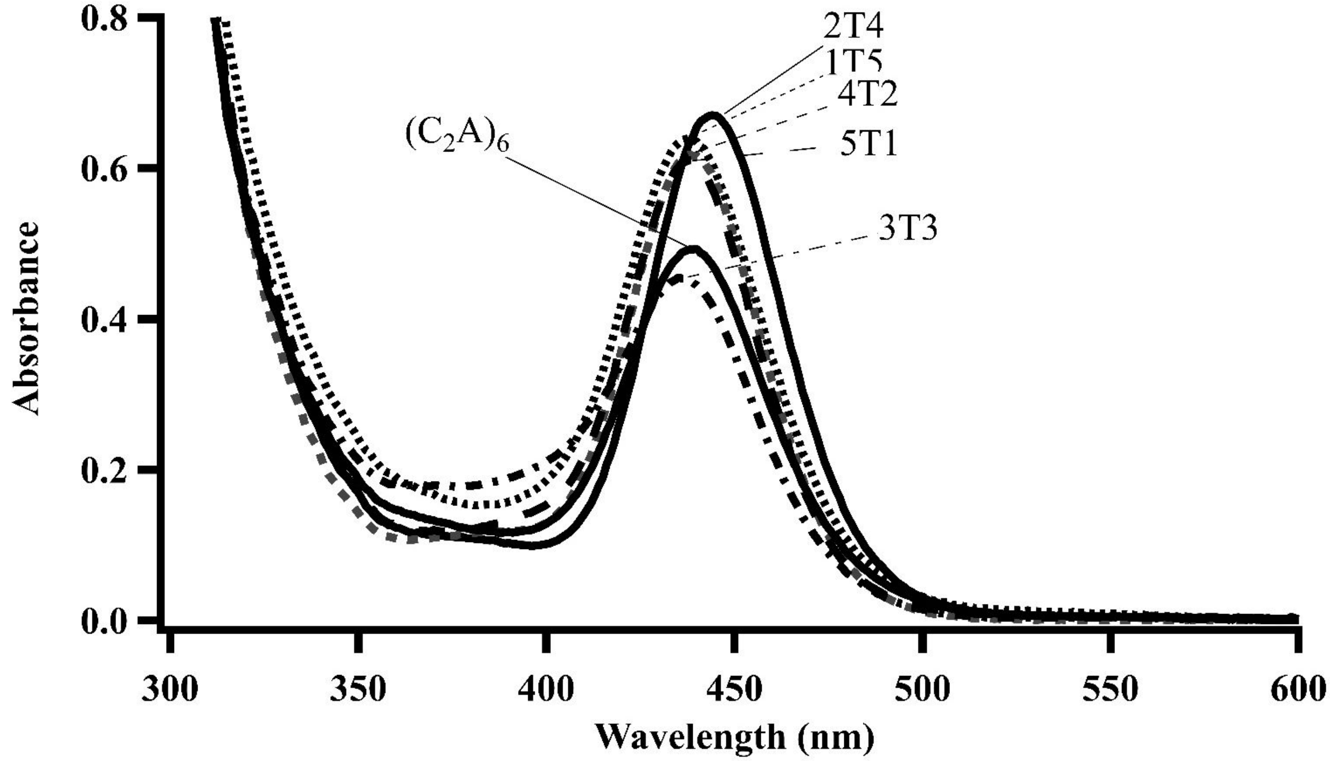


Figure S1: Absorption spectra of $(C_2A)_6$ and xTy-silver cluster chromophores. The DNA concentrations are 30 μM and 240 μM $Ag^+$ and 120 μM $BH_4^-$. Similar spectra indicate similar binding sites for the silver cluster adducts in the homogeneous $(C_2A)_6$ vs the interrupted xTy strands.

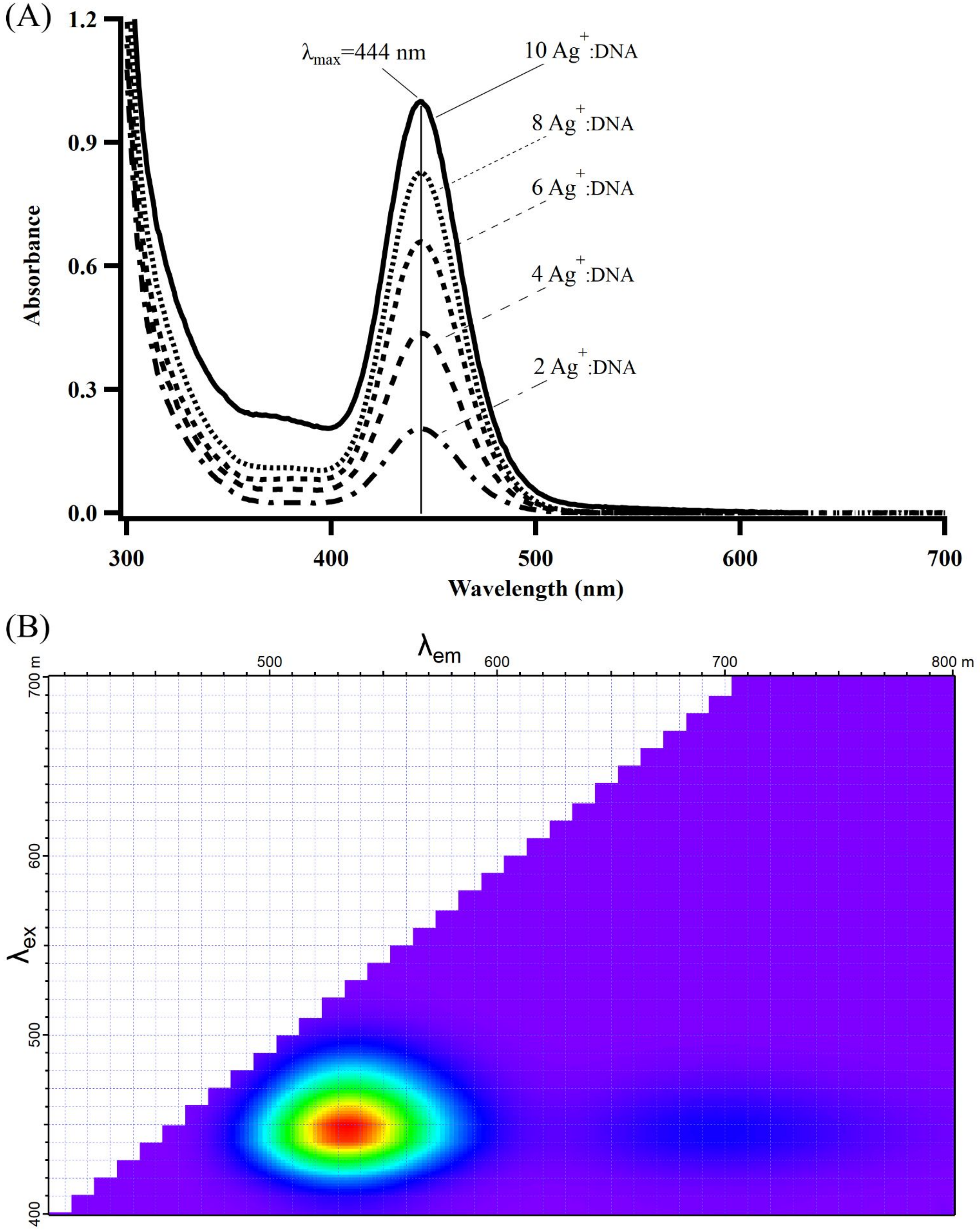


Figure S2: (A) Absorption spectra of 30 μM 2T4 with initial 2, 4, 6, 8, and 10 equivalents of $Ag^{+}$. Only the 444 nm absorption band develops over this concentration range, and the vertical line shows that the $\lambda_{max}$ is constant. (B) Contour plot of the excitation and emission spectra of $2T4/Ag_{10}^{6+}$. The consistent emission spectra suggest that a single fluorophore is favored.

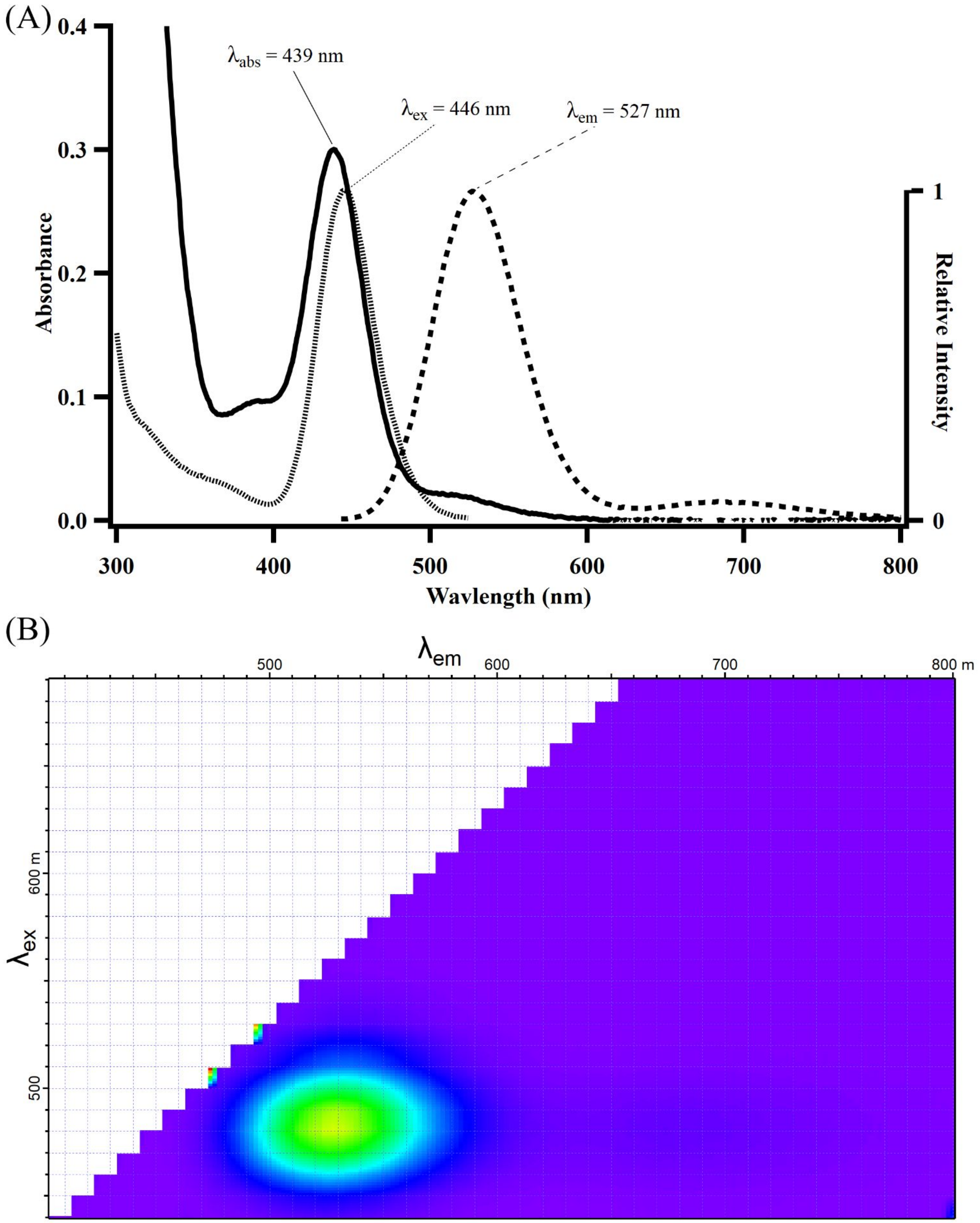


Figure S3: (A) Absorption (left axis, solid) and excitation (right axis, dotted)/emission (right axis, dashed) spectra of 2+4/$Ag_{10}^{6+}$. Similar spectra to 2T4/$Ag_{10}^{6+}$ suggest that $(C_2A)_2$ and $(C_2A)_4$ assemble to form a similar coordination site. (B) Contour plot of the excitation and emission spectra of 2+4/$Ag_{10}^{6+}$. The consistent emission spectra suggest that a single fluorophore is favored.

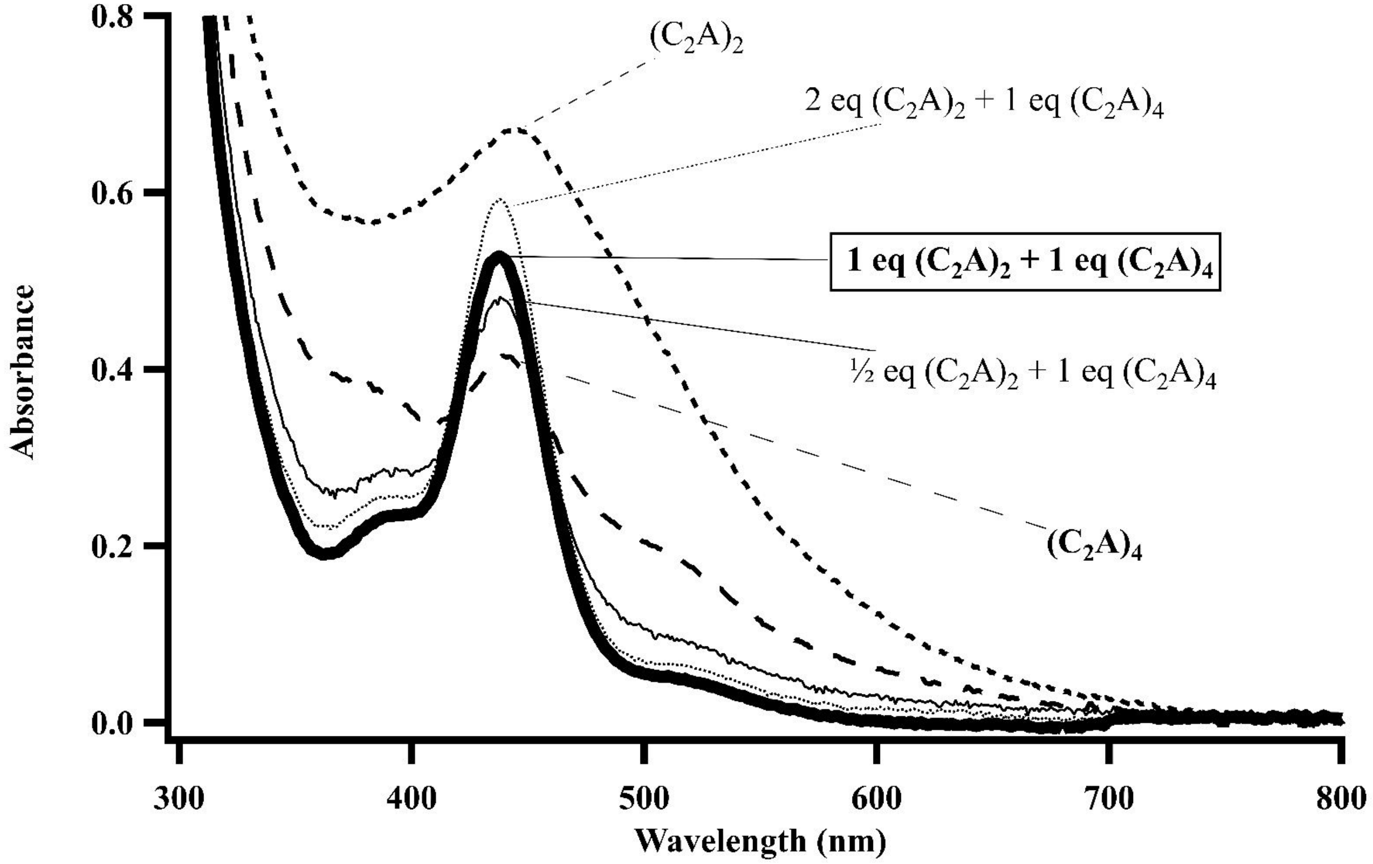


Figure S4: Absorption spectra of silver adducts with 30 µM $(C_2A)_2$ alone (short dashed), 30 µM $(C_2A)_4$ alone (long dashed), 30 µM $(C_2A)_4$ + ½ equivalent $(C_2A)_2$ (thin solid), 30 µM $(C_2A)_4$ + 1 equivalent $(C_2A)_2$ (heavy solid), and 30 µM $(C_2A)_4$ + 2 equivalent $(C_2A)_2$ (dotted). A heterodimer $(C_2A)_2$ + $(C_2A)_4$ is supported because distinct spectra are observed for $(C_2A)_2$ alone and $(C_2A)_4$ alone and because stronger absorption is favored with larger relative amounts of $(C_2A)_2$ vs $(C_2A)_4$.

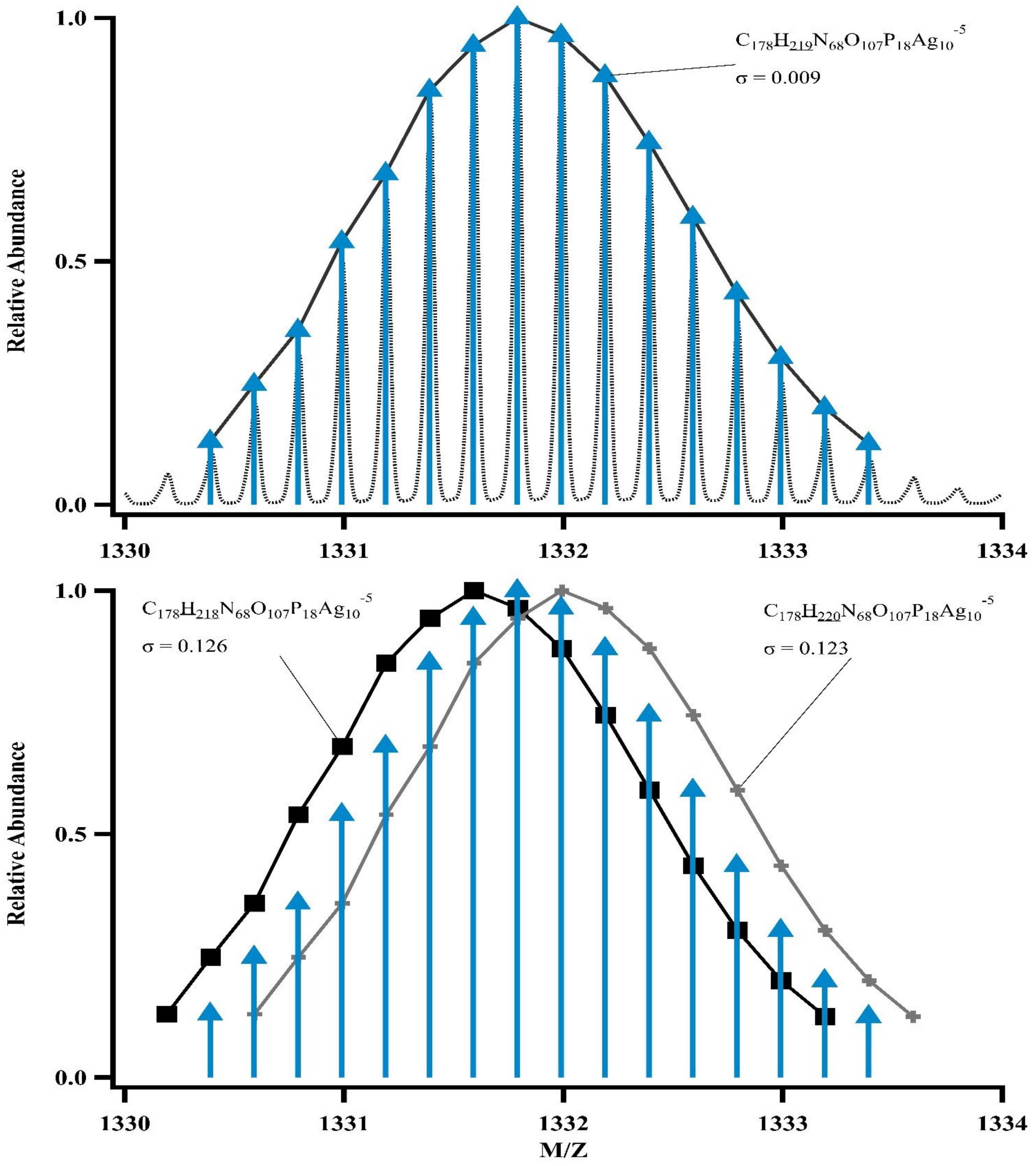


**Figure S5:** (Top) Isotope model for the -5 charge state based on the molecular formula $C_{178}H_{219}O_{107}N_{68}P_{18}Ag_{10}^{-5}$. The standard deviation for the intensity distribution (σ) is based on the formula

$$\sigma = \sqrt{\frac{\Sigma_N (I_{measured} - I_{model})^2}{N-1}}$$

where $I_{measured}$ is the measured intensity, $I_{model}$ is the predicted intensity, and $N$ is the number of peaks in the distribution.[1] For this envelop, σ = 0.009. (Bottom) The isotope model for based on the formulas $C_{178}H_{218}O_{107}N_{68}P_{18}Ag_{10}^{-5}$ (black squares) and $C_{178}H_{220}O_{107}N_{68}P_{18}Ag_{10}^{-5}$ (gray crosses). Underlined subscripts emphasize the numbers of $H^+$, relative to the formula used for part (A), respectively

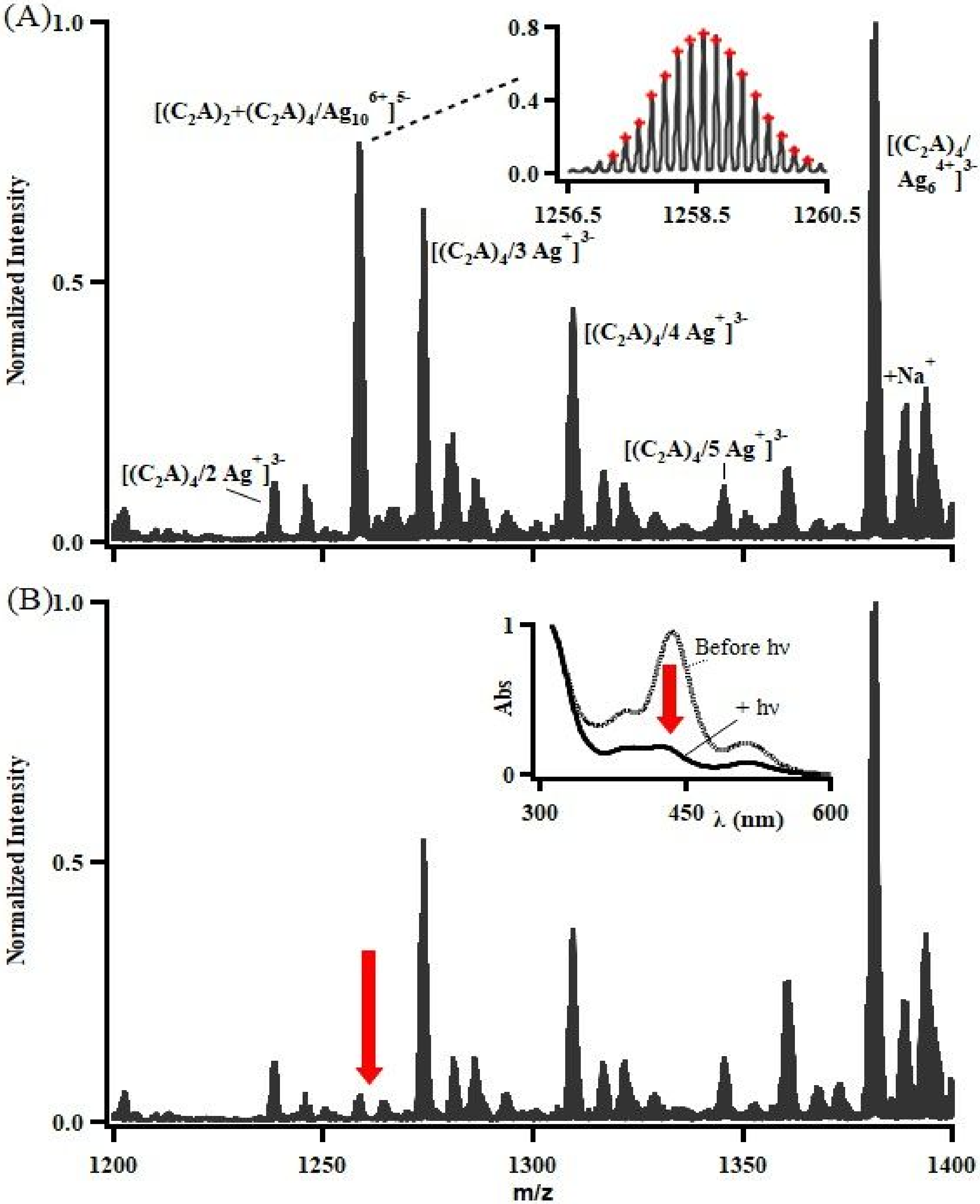


Figure S6: Mass:charge spectra of $[2+4/Ag_{10}^{6+}]^{-4}$ before (A) and after (B) irradiation for 1 hr. at 428 nm. The inset in (A) show the isotope distributions for this cluster that correspond to the formula $[C_{168}H_{208}N_{66}O_{98}P_{16}(Ag_{10}^{6+})]^{-4}$. Additional peaks in the spectra are due to $(C_2A)_4$-$Ag^+$ and $Ag_6^{4+}$ adducts and are not impacted by irradiation (Table S4).

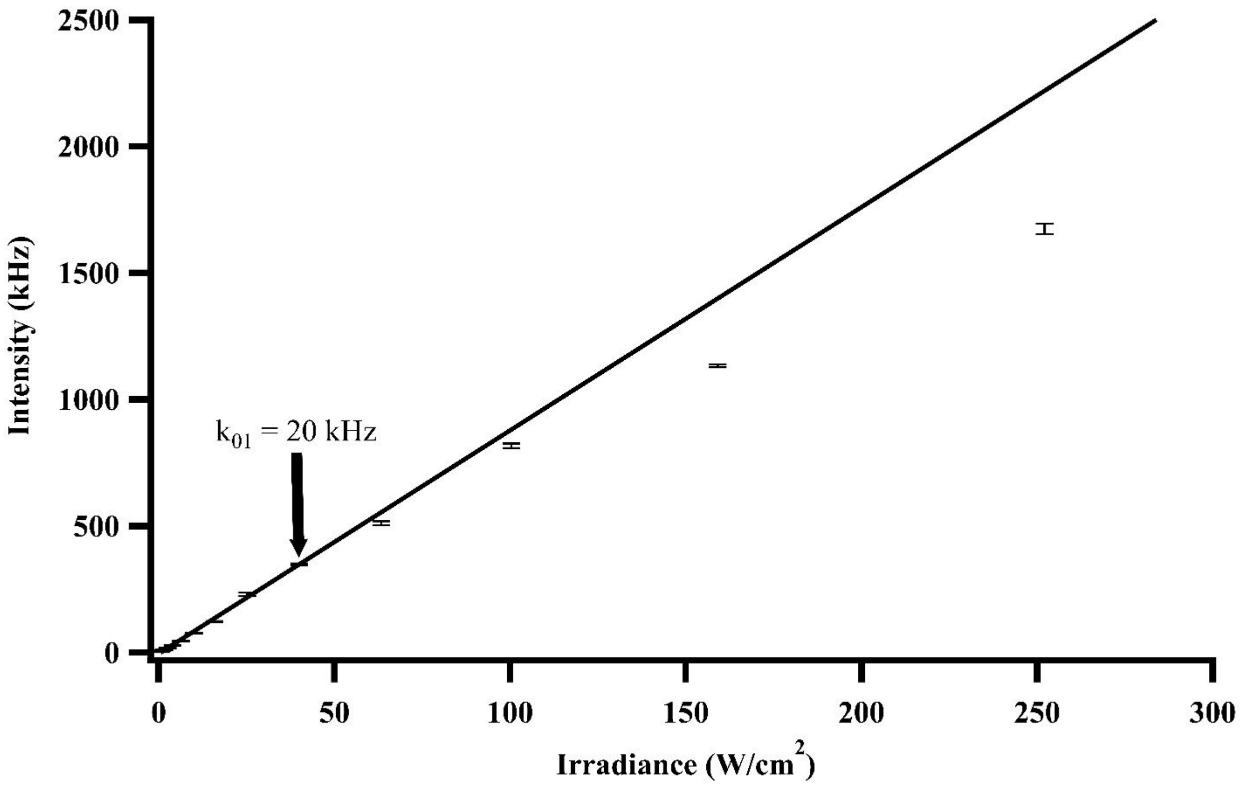


Fig. S7: Fluorescence intensity vs. irradiance for $2T4/Ag_{10}^{6+}$. The first 11 measurements were used to create a linear fit, and subsequent measurements deviate from linearity at ~45 W/cm$^2$.

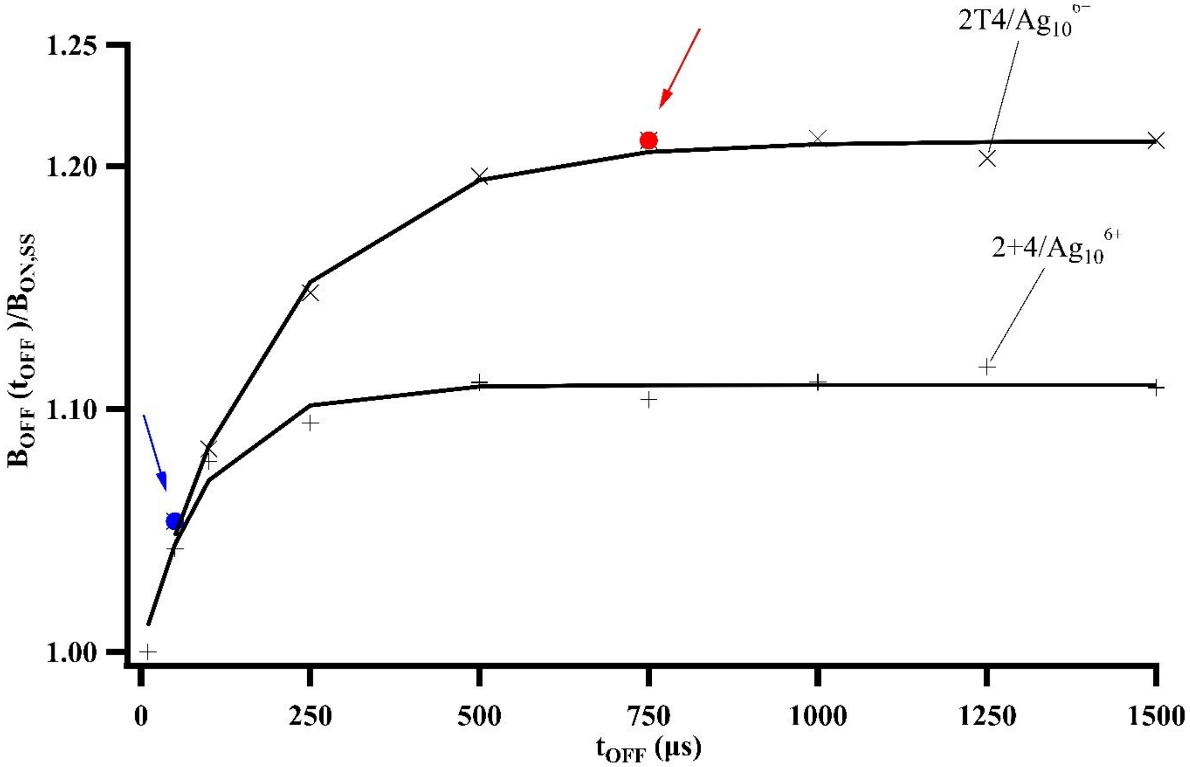


Figure S8: Normalized intensities for $2T4/Ag_{10}^{6+}$ (X) and $2{+}4/Ag_{10}^{6+}$ (+). These were fit with Equation 8 to determine $k_{ON}$ = 1084 ± 47 Hz and $k_{OFF}$. = 5150 ± 250 Hz for $2T4/Ag_{10}^{6+}$ and $k_{ON}$ = 1130 ± 140 Hz and $k_{OFF}$. = 10300 ± 1400 Hz for $2{+}4/Ag_{10}^{6+}$. The red and blue arrows indicate the respective decays in Figue 2A for $2T4/Ag_{10}^{6+}$.

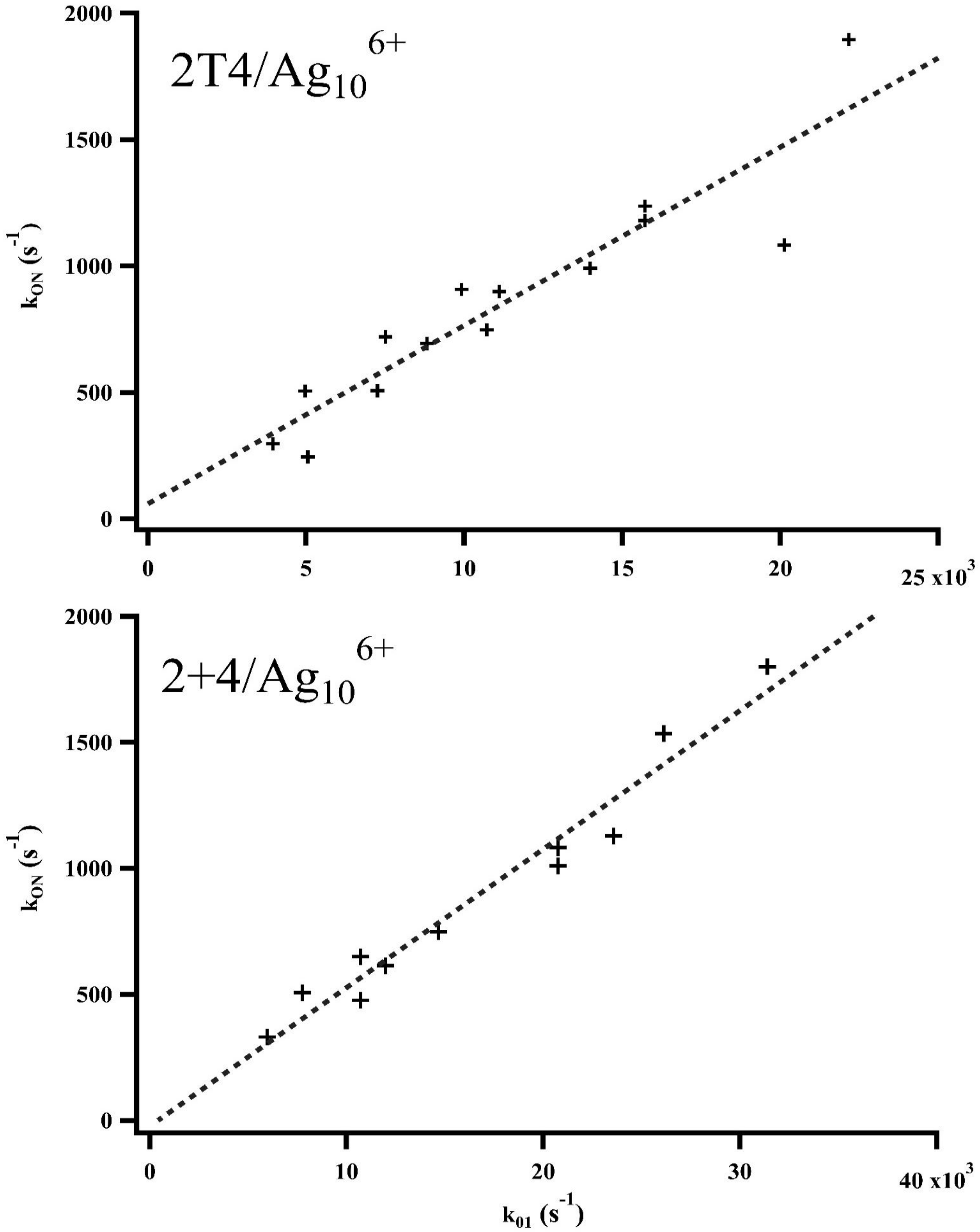


Figure S9: Dependence of $k_{ON}$ with the excitation rate $k_{01}$. The data are fit with the equation $k_{ON} = k_{01}\ \phi_{12}$, where $k_{01} = \sigma I$ and I is the irradiance ($W/cm^2$) and $\sigma$ is the absorption cross-section ($cm^2$). Linear fits yielded $\phi_{12} = 7 \pm 1\%$ with an intercept of $60 \pm 100$ for $2T4/Ag_{10}^{6+}$ and $\phi_{12} = 5.5 \pm 0.5\%$ with an intercept of $-23 \pm 70$ for $2{+}4/Ag_{10}^{6+}$.

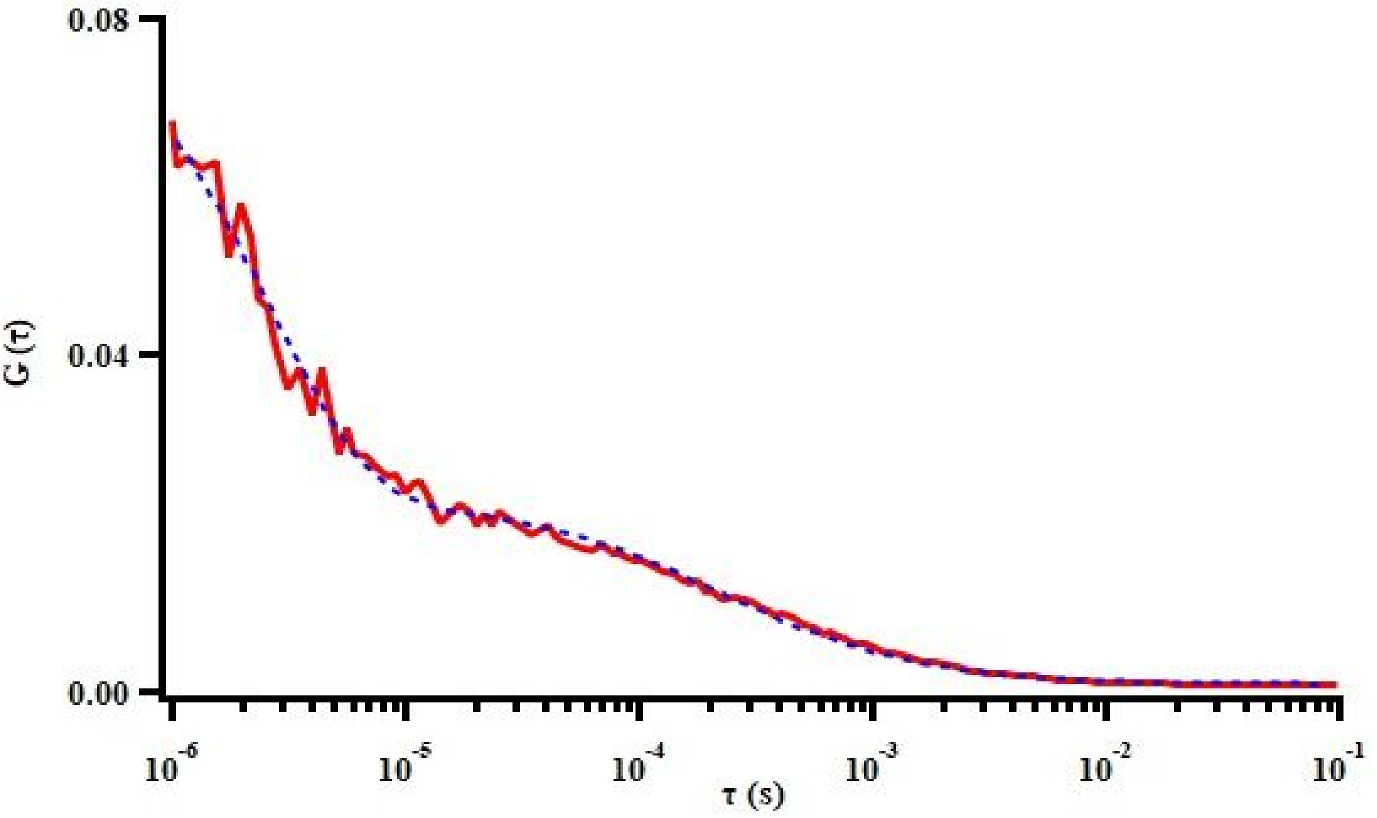


Figure S10: Fluorescence correlation functions for the $(C_2A)_6/Ag_{10}^{6+}$ (solid red trace). The fit (dotted blue trace) gives N = 46 molecules, $t_d$ = 223 μs, F = 75%, and $t_{ES}$ = 0.75 μs (see Equ. 1-3). The structure factor z was determined using Rhodamine 110 to be 8.6.

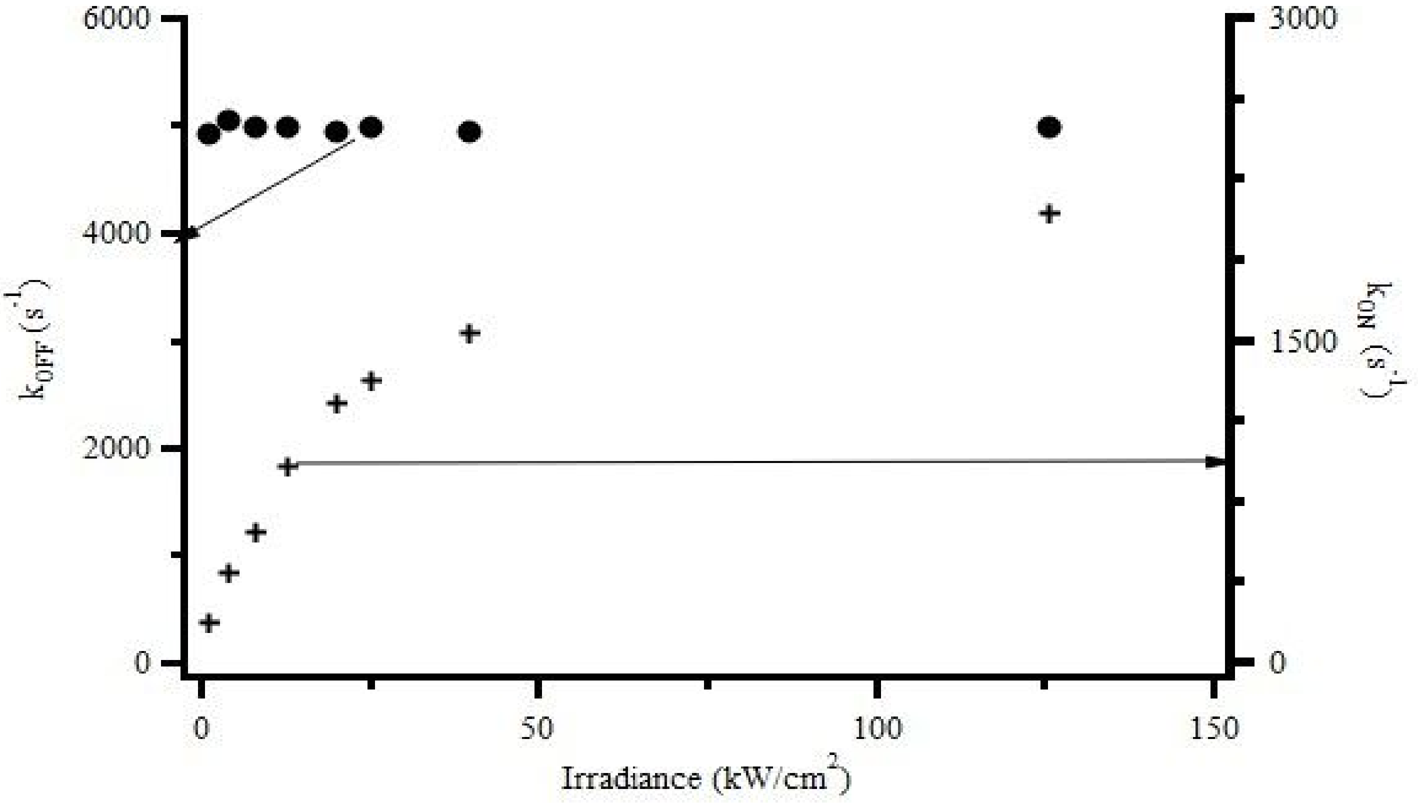


Figure S11: Dependence of $k_{OFF}$ (left axis) and $k_{ON}$ (right axis) with the irradiance. The consistent $k_{OFF}$ values suggest that laser absorption by the intermediate state is minor.

Table S1: Spectral and Fluorescence Parameters for xTy Derivatives of $(C_2A)_6$[a]

| Sequence | $\lambda_{abs}/\lambda_{em}$ (nm) | $\phi_F$ | $\tau_F$ (ns) |
|---|---|---|---|
| **$(CCA)_6$**<br><br>CCA CCA CCA CCA CCA CCA | 439/536 | 0.15 ± 0.04 | 2.1 ± 0.2 |
| **2T4**<br><br>CCA CCA **T** CCA CCA CCA CCA | 445/535 | 0.20 ± 0.01 | 1.57 ± 0.03 |
| **2+4**<br><br>CCA CCA + CCA CCA CCA CCA | 439 | 0.08 ± 0.01 | 1.3 ± 0.3 |
| **1T5**<br><br>CCA **T** CCA CCA CCA CCA CCA | 438/531 | 0.19 ± 0.04 | 2.2 |
| **3T3**<br><br>CCA CCA CCA **T** CCA CCA CCA | 437/531 | 0.07 ± 0.01 | 1.8 |
| **4T2**<br><br>CCA CCA CCA CCA **T** CCA CCA | 439/531 | 0.11 ± 0.02 | 1.4 |
| **5T1**<br><br>CCA CCA CCA CCA CCA **T** CCA | 438/532 | 0.15 ± 0.03 | 1.8 |

[a] $\lambda_{abs}/\lambda_{em}$ are the absorption and emission maxima, respectively, $\phi_F$ is the fluorescence quantum yield, $\tau_F$ is the fluorescence lifetime.

Table S2: Summary of Mass:Charge data for 2T4/$Ag_{10}^{6+}$ (top) and 2+4/$Ag_{10}^{6+}$ (bottom) [a]

| $[2T4/ Ag_{10}^{6+}]^{-4}$ | | | $[2T4/ Ag_{10}^{6+}]^{-5}$ | | | $[2T4/ Ag_{10}^{6+}]^{-6}$ | | |
|---|---|---|---|---|---|---|---|---|
| **Experiment** | **Predicted** | **Δ (PPM)** | **Experiment** | **Predicted** | **Δ (PPM)** | **Experiment** | **Predicted** | **Δ (PPM)** |
| 1663.2361 | 1663.2421 | 3.61 | 1330.3893 | 1330.3921 | 2.10 | 1108.488 | 1108.4921 | 3.70 |
| 1663.4866 | 1663.4919 | 3.19 | 1330.5891 | 1330.5919 | 2.10 | 1108.6549 | 1108.6587 | 3.43 |
| 1663.7363 | 1663.7422 | 3.55 | 1330.7891 | 1330.7921 | 2.25 | 1108.8220 | 1108.8254 | 3.07 |
| 1663.9862 | 1663.9921 | 3.55 | 1330.9891 | 1330.9921 | 2.25 | 1108.9888 | 1108.9921 | 2.98 |
| 1664.2368 | 1664.2423 | 3.30 | 1331.1892 | 1331.1923 | 2.33 | 1109.1553 | 1109.1589 | 3.25 |
| 1664.4863 | 1664.4923 | 3.60 | 1331.3892 | 1331.3922 | 2.25 | 1109.3220 | 1109.3256 | 3.25 |
| 1664.7368 | 1664.7424 | 3.36 | 1331.5891 | 1331.5924 | 2.48 | 1109.4889 | 1109.4923 | 3.06 |
| 1664.9863 | 1664.9924 | 3.66 | 1331.7892 | 1331.7924 | 2.40 | 1109.6550 | 1109.6591 | 3.69 |
| 1665.2362 | 1665.2426 | 3.84 | 1331.9891 | 1331.9926 | 2.63 | 1109.8221 | 1109.8258 | 3.33 |
| 1665.4865 | 1665.4927 | 3.72 | 1332.1892 | 1332.1926 | 2.55 | 1109.9888 | 1109.9926 | 3.42 |
| 1665.7363 | 1665.7428 | 3.90 | 1332.3893 | 1332.3927 | 2.55 | 1110.1553 | 1110.1593 | 3.60 |
| 1665.9867 | 1665.9930 | 3.78 | 1332.5895 | 1332.5928 | 2.48 | 1110.3223 | 1110.3260 | 3.33 |
| 1666.2367 | 1666.2432 | 3.90 | 1332.7897 | 1332.7930 | 2.48 | 1110.4890 | 1110.4928 | 3.42 |
| 1666.4865 | 1666.4933 | 4.08 | 1332.9899 | 1332.9930 | 2.33 | 1110.6558 | 1110.6597 | 3.51 |
| 1666.7382 | 1666.7435 | 3.18 | 1333.1902 | 1333.1932 | 2.25 | 1110.8224 | 1110.8264 | 3.60 |
| 1666.9885 | 1666.9938 | 3.18 | 1333.3905 | 1333.3934 | 2.17 | 1110.9907 | 1110.9933 | 2.34 |
| | $\bar{\Delta}$ | 3.6 | | $\bar{\Delta}$ | 2.4 | | $\bar{\Delta}$ | 3.3 |
| | $\Delta_{\sigma}$ | 0.3 | | $\Delta_{\sigma}$ | 0.2 | | $\Delta_{\sigma}$ | 0.3 |

| $[2+4/ Ag_{10}^{6+}]^{-4}$ | | | $[2+4/ Ag_{10}^{6+}]^{-5}$ | | |
|---|---|---|---|---|---|
| **Experiment** | **Predicted** | **Δ (PPM)** | **Experiment** | **Predicted** | **Δ (PPM)** |
| 1571.7325 | 1571.7416 | 5.79 | 1257.1862 | 1257.1917 | 4.37 |
| 1571.9861 | 1571.9913 | 3.31 | 1257.3899 | 1257.3915 | 1.27 |
| 1572.2393 | 1572.2417 | 1.53 | 1257.5892 | 1257.5918 | 2.07 |
| 1572.4878 | 1572.4916 | 2.42 | 1257.7891 | 1257.7916 | 1.99 |
| 1572.7373 | 1572.7417 | 2.80 | 1257.9897 | 1257.9918 | 1.67 |
| 1572.9879 | 1572.9917 | 2.42 | 1258.1891 | 1258.1918 | 2.15 |
| 1573.2386 | 1573.2419 | 2.10 | 1258.3899 | 1258.3920 | 1.67 |
| 1573.4882 | 1573.4919 | 2.35 | 1258.5898 | 1258.5919 | 1.67 |
| 1573.7384 | 1573.7421 | 2.35 | 1258.7897 | 1258.7921 | 1.91 |
| 1573.9891 | 1573.9922 | 1.97 | 1258.9899 | 1258.9922 | 1.83 |
| 1574.2379 | 1574.2423 | 2.80 | 1259.1896 | 1259.1923 | 2.14 |
| 1574.4882 | 1574.4924 | 2.67 | 1259.3907 | 1259.3923 | 1.27 |
| 1574.7406 | 1574.7426 | 1.27 | 1259.5901 | 1259.5925 | 1.91 |
| 1574.9897 | 1574.9928 | 1.97 | 1259.7904 | 1259.7926 | 1.75 |
| 1575.2472 | 1575.2429 | 2.73 | 1259.9910 | 1259.9928 | 1.43 |
| 1575.4930 | 1575.4932 | 0.13 | 1260.1897 | 1260.1930 | 2.62 |
| | $\bar{\Delta}$ | 2 | | $\bar{\Delta}$ | 2.0 |
| | $\Delta_{\sigma}$ | 1 | | $\Delta_{\sigma}$ | 0.7 |

[a] Δ is the difference between the Experiment and Predicted M/Z values in ppm $\left(\frac{|Exp - Pred|}{Pred} x10^6\right)$, $\bar{\Delta}$ is the average difference, and **Δσ** is the standard deviation of the differences.

Table S3: Mass:Charge data for Additional Peaks in Figure 1b[a]

$[2T4]^{-5}$

| Experiment | Predicted | Δ (PPM) |
|---|---|---|
| 1116.790 | 1116.791 | 0.8 |
| 1116.987 | 1116.991 | 3.6 |
| 1117.188 | 1117.192 | 3.8 |
| 1117.387 | 1117.392 | 4.4 |
| 1117.590 | 1117.593 | 3.0 |
| 1117.790 | 1117.793 | 2.6 |
| 1117.990 | 1117.994 | 3.4 |
| | $\bar{\Delta}$ | 3.1 |
| | $\Delta_\sigma$ | 1.2 |

$[2T4/Ag^{+}]^{-5}$

| Experiment | Predicted | Δ (PPM) |
|---|---|---|
| 1137.967 | 1137.970 | 2.7 |
| 1138.166 | 1138.171 | 4.1 |
| 1138.367 | 1138.371 | 3.3 |
| 1138.567 | 1138.571 | 4.0 |
| 1138.768 | 1138.772 | 3.3 |
| 1138.968 | 1138.972 | 3.3 |
| 1139.168 | 1139.172 | 3.7 |
| 1139.368 | 1139.373 | 4.6 |
| 1139.568 | 1139.573 | 4.8 |
| | $\bar{\Delta}$ | 3.8 |
| | $\Delta_\sigma$ | 0.7 |

$[2T4/Ag_6^{4+}]^{-5}$

| Experiment | Predicted | Δ (PPM) |
|---|---|---|
| 1244.671 | 1244.667 | 2.7 |
| 1244.871 | 1244.867 | 3.4 |
| 1245.071 | 1245.067 | 3.1 |
| 1245.271 | 1245.268 | 3.0 |
| 1245.471 | 1245.468 | 2.8 |
| 1245.672 | 1245.668 | 2.8 |
| 1245.872 | 1245.868 | 2.7 |
| 1246.072 | 1246.068 | 2.6 |
| 1246.272 | 1246.268 | 3.3 |
| 1246.472 | 1246.468 | 2.9 |
| 1246.672 | 1246.669 | 2.9 |
| 1246.872 | 1246.869 | 2.9 |
| 1247.073 | 1247.070 | 2.5 |
| 1247.273 | 1247.268 | 3.6 |
| | $\bar{\Delta}$ | 2.9 |
| | $\Delta_\sigma$ | 0.3 |

$[2T4]^{-4}$

| Experiment | Predicted | Δ (PPM) |
|---|---|---|
| 1396.233 | 1396.240 | 5.5 |
| 1396.491 | 1396.491 | 0.2 |
| 1396.739 | 1396.742 | 2.0 |
| 1396.987 | 1396.992 | 4.1 |
| 1397.237 | 1397.243 | 4.3 |
| 1397.492 | 1397.494 | 1.1 |
| 1397.745 | 1397.744 | 0.8 |
| 1397.989 | 1397.995 | 3.9 |
| | $\bar{\Delta}$ | 2.7 |
| | $\Delta_\sigma$ | 1.9 |

$[2T4/Ag_6^{4+}]^{-4}$

| Experiment | Predicted | Δ (PPM) |
|---|---|---|
| 1556.078 | 1556.091 | 7.9 |
| 1556.336 | 1556.341 | 3.3 |
| 1556.587 | 1556.591 | 2.4 |
| 1556.837 | 1556.841 | 3.0 |
| 1557.087 | 1557.091 | 3.0 |
| 1557.337 | 1557.341 | 3.1 |
| 1557.587 | 1557.591 | 3.1 |
| 1557.837 | 1557.842 | 3.0 |
| 1558.087 | 1558.092 | 3.1 |
| 1558.337 | 1558.342 | 3.3 |
| 1558.588 | 1558.592 | 3.0 |
| 1558.837 | 1558.843 | 3.4 |
| 1559.084 | 1559.093 | 5.7 |
| 1559.339 | 1559.343 | 2.4 |
| | $\bar{\Delta}$ | 3.6 |
| | $\Delta_\sigma$ | 1.5 |

[a] Δ is the difference between the Experiment and Predicted M/Z values in ppm $\left(\frac{|Exp - Pred|}{Pred} x10^6\right)$, $\bar{\Delta}$ is the average difference, and $\Delta\boldsymbol{\sigma}$ is the standard deviation of the differences.

Table 4S: Mass:Charge data for Additional Peaks in Figure 6S[a]

| $[(C_2A)_4/2Ag^+]^{-3}$ | | |
|---|---|---|
| **Experiment** | **Predicted** | **Δ (PPM)** |
| 1237.139 | 1237.136 | 2.3 |
| 1237.473 | 1237.468 | 3.9 |
| 1237.806 | 1237.8 | 4.8 |
| 1238.14 | 1238.135 | 3.8 |
| 1238.474 | 1238.469 | 3.3 |
| 1238.807 | 1238.801 | 4.8 |
| 1239.141 | 1239.139 | 1.5 |
| | $\bar{\Delta}$ | 3.2 |
| | $\Delta_\sigma$ | 1.3 |

| $[(C_2A)_4/3Ag^+]^{-3}$ | | |
|---|---|---|
| **Experiment** | **Predicted** | **Δ (PPM)** |
| 1272.430 | 1272.438 | 6.6 |
| 1272.770 | 1272.772 | 2.0 |
| 1273.104 | 1273.105 | 1.0 |
| 1273.435 | 1273.439 | 3.0 |
| 1273.770 | 1273.772 | 2.0 |
| 1274.105 | 1274.106 | 1.2 |
| 1274.436 | 1274.440 | 3.0 |
| | $\bar{\Delta}$ | 2.9 |
| | $\Delta_\sigma$ | 2.3 |

| $[(C_2A)_4/4Ag^+]^{-3}$ | | |
|---|---|---|
| **Experiment** | **Predicted** | **Δ (PPM)** |
| 1307.740 | 1307.737 | 2.1 |
| 1308.076 | 1308.071 | 3.8 |
| 1308.401 | 1308.404 | 2.4 |
| 1308.738 | 1308.738 | 0.4 |
| 1309.069 | 1309.071 | 1.5 |
| 1309.402 | 1309.405 | 2.2 |
| 1309.736 | 1309.738 | 1.5 |
| 1310.070 | 1310.072 | 1.7 |
| 1310.402 | 1310.406 | 2.6 |
| 1310.737 | 1310.739 | 1.7 |
| 1311.070 | 1311.073 | 2.3 |
| 1311.400 | 1311.407 | 5.2 |
| | $\bar{\Delta}$ | 2.3 |
| | $\Delta_\sigma$ | 1.2 |

| $[(C_2A)_4/5Ag^+]^{-3}$ | | |
|---|---|---|
| **Experiment** | **Predicted** | **Δ (PPM)** |
| 1344.0422 | 1344.039 | 2.2 |
| 1344.375 | 1344.368 | 5.3 |
| 1344.709 | 1344.703 | 4.5 |
| 1345.0419 | 1345.038 | 3.3 |
| 1345.3757 | 1345.37 | 4.1 |
| 1345.709 | 1345.704 | 3.7 |
| 1346.0426 | 1346.038 | 3.3 |
| 1346.376 | 1346.372 | 3.1 |
| 1346.7096 | 1346.709 | 0.1 |
| 1347.0432 | 1347.041 | 1.6 |
| 1347.377 | 1347.377 | 0.1 |
| | $\bar{\Delta}$ | 2.9 |
| | $\Delta_\sigma$ | 1.7 |

| $[(C_2A)_4/Ag_6^{4+}]^{-3}$ | | |
|---|---|---|
| **Experiment** | **Predicted** | **Δ (PPM)** |
| 1379.6735 | 1379.674 | 0.4 |
| 1380.006 | 1380.0081 | 1.5 |
| 1380.3385 | 1380.3409 | 1.7 |
| 1380.6725 | 1380.6748 | 1.7 |
| 1381.0059 | 1381.0078 | 1.4 |
| 1381.3394 | 1381.3416 | 1.6 |
| 1381.673 | 1381.6748 | 1.3 |
| 1382.0067 | 1382.0084 | 1.2 |
| 1382.3402 | 1382.3419 | 1.2 |
| 1382.6737 | 1382.6755 | 1.3 |
| 1383.0082 | 1383.009 | 0.6 |
| 1383.3425 | 1383.3428 | 0.2 |
| 1383.6774 | 1383.6765 | 0.7 |
| 1379.6735 | 1379.674 | 0.4 |
| | $\bar{\Delta}$ | 1.1 |
| | $\Delta_\sigma$ | 0.5 |

| $[(C_2A)_4/Ag_6^{4+}/Na^+]^{-3}$ | | |
|---|---|---|
| **Experiment** | **Predicted** | **Δ (PPM)** |
| 1387.001 | 1387.001 | 0.1 |
| 1387.339 | 1387.335 | 2.8 |
| 1387.668 | 1387.668 | 0.4 |
| 1388.002 | 1388.002 | 0.1 |
| 1388.333 | 1388.335 | 1.3 |
| 1388.668 | 1388.669 | 0.8 |
| 1389.002 | 1389.002 | 0.1 |
| 1389.335 | 1389.336 | 0.8 |
| 1389.669 | 1389.669 | 0.1 |
| 1390.004 | 1390.003 | 0.5 |
| 1390.340 | 1390.336 | 2.4 |
| 1390.671 | 1390.670 | 0.8 |
| | $\bar{\Delta}$ | 0.9 |
| | $\Delta_\sigma$ | 0.9 |

[a] Δ is the difference between the Experiment and Predicted M/Z values in ppm $\left(\frac{|Exp - Pred|}{Pred} x10^6\right)$, $\bar{\Delta}$ is the average difference, and Δ**σ** is the standard deviation of the differences.

References:

1. Comeau, A. N.; Liu, J.; Khadka, C. B.; Corrigan, J. F.; Konermann, L. Nanocluster Isotope Distributions Measured by Electrospray Time-of-Flight Mass Spectrometry. *Anal. Chem.* **2012**, *85*, 1200-1207.
